# Integrative analysis of mortality risk in SFTS using machine learning and genetic approaches

**DOI:** 10.1128/msphere.00831-25

**Published:** 2026-06-16

**Authors:** Helin Zha, Lianzi Wang, Haoyang Sun, Jie Li, Yajing Wang

**Affiliations:** 1 Department of Clinical Laboratory, The First Affiliated Hospital of Anhui Medical University 36639, Hefei, China; 2 Faculty of Innovation Engineering, Macau University of Science and Technology, Taipa, Macau; 3 Department of Rehabilitation, Hefei Third People's Hospital, Hefei, Anhui, China; Duke-NUS Medical School, Singapore, Singapore

**Keywords:** severe fever with thrombocytopenia syndrome, mortality prediction, machine learning, extreme gradient boosting, Mendelian randomization, metabolic pathways

## Abstract

This study presents a novel approach to predicting mortality risk in patients with severe fever with thrombocytopenia syndrome (SFTS) by integrating machine learning models and genetic analysis techniques. Utilizing clinical data from 107 confirmed cases, we developed several predictive models and identified the extreme gradient boosting (XGBoost) algorithm as the most effective, achieving an area under the curve (AUC) of 0.950. Further analysis was performed based on the 15 robust features selected and performance-optimized through the Boruta algorithm, integrating Shapley additive explanations (SHAP) plots to identify key variables such as IL-6, TNF-α, PLR, and AST. While Mendelian randomization did not reveal definitive causal pathways across all examined factors, notable associations were observed in specific metabolic-inflammatory pathways. These findings offer a foundation for enhanced predictive accuracy and mechanistic understanding of SFTS mortality.

**IMPORTANCE:**

Severe fever with thrombocytopenia syndrome (SFTS) is a tick-borne viral disease with high mortality, posing a major threat to public health in East Asia. This study develops a machine learning (ML) model to quickly predict SFTS patients’ mortality risk using routine clinical tests, helping doctors stratify risks early. Moreover, by exploring causal pathways between bodily indicators and disease outcomes, it provides new clues for understanding why SFTS becomes severe, which could guide better treatments in the future.

## INTRODUCTION

Severe fever with thrombocytopenia syndrome (SFTS) is a newly identified infectious disease caused by the SFTS virus, predominantly endemic to Asian countries such as China, South Korea, and Japan (1–4). Since its first report in China in 2009, the disease has attracted widespread attention due to its high fatality rate, which ranges from approximately 12% to 30% (5, 6). The clinical manifestations of SFTS include high fever, thrombocytopenia, and multiple organ dysfunction, and in severe cases, it can lead to patient mortality (7, 8). Due to the complex pathogenesis of SFTS, predicting disease progression and mortality risk in clinical settings remains challenging (9, 10). Currently, the pathogenic mechanisms and risk prediction of SFTS are not fully understood, limiting the development of effective therapeutic strategies and disease management approaches (11).

Current research on SFTS has primarily focused on virological characteristics, epidemiological investigations, and descriptions of clinical features, whereas studies aimed at predicting patient mortality risk remain relatively scarce (5, 12, 13). Existing predictive models mainly rely on statistical methods such as logistic regression (LR), which assess clinical indicators (e.g., white blood cell count and platelet count) in SFTS patients (10). While these methods can predict disease outcomes to some extent, they have limitations in data handling and result interpretation (14). For example, such models often overlook potential nonlinear relationships and interactions between variables (15), resulting in insufficient predictive accuracy and stability. Moreover, traditional models rarely elucidate the disease’s biological mechanisms, which hinders a deeper understanding of the pathophysiological processes and causes of SFTS mortality (16).

In recent years, machine learning (ML) techniques have been widely applied in healthcare, particularly demonstrating significant potential in disease diagnosis and risk prediction (17, 18). ML models such as random forest (RF), support vector machine (SVM), and extreme gradient boosting (XGBoost) have been successfully used for predicting various diseases (19). These models can handle complex data sets and identify nonlinear relationships and interaction effects among variables (20, 21). For example, the XGBoost algorithm has been widely favored in clinical data analysis due to its excellent classification performance, high processing speed, and strong interpretability (22). These ML approaches improve prediction accuracy and provide new perspectives and tools for disease management and therapeutic decision-making (23).

Mendelian randomization (MR) is a method that uses genetic variants as instrumental variables to assess causal relationships between exposures and outcomes (24). By minimizing the influence of confounding factors, this approach overcomes the limitations of traditional observational studies and enhances the credibility of research findings (25, 26). In medical research, MR can be used to validate known biomarkers and uncover novel pathological mechanisms (27). Especially in complex metabolic, inflammatory, and immune pathways, MR offers a genetic-level approach to exploring potential causal relationships (28, 29), which is of great importance for understanding disease biology and developing new treatments.

This study combined cutting-edge ML techniques with the MR approach to construct an efficient mortality risk prediction model for SFTS and explore causal pathways among key metabolic, inflammatory, and immune factors to reveal their synergistic mechanisms. The scientific significance of this research lies in its provision of a novel, data-driven strategy to improve the accuracy of SFTS mortality risk prediction. Furthermore, by identifying critical biological variables and their interactions within the pathological process, this study offers new insights into the underlying mechanisms of SFTS-related mortality. Clinically, the implementation of an effective predictive model holds substantial promise for enabling real-time risk assessment, facilitating early clinical intervention, and informing personalized treatment strategies for high-risk patients. These contributions may ultimately improve patient outcomes, support public health decision-making, and mitigate the societal and economic burden associated with SFTS.

## RESULTS

### Evaluation of discriminative power and fitting performance across four ML models

We systematically evaluated and compared the performance of four commonly used classification algorithms—LR, RF, XGBoost, and SVM—in predicting mortality risk among patients with SFTS. A summary of the model performance metrics is presented in Table 1. The results showed that the XGBoost model and random forest models both demonstrated high and comparable predictive performance. The XGBoost model achieved an area under the curve (AUC) of 0.950, an accuracy of 0.879, an F1 score of 0.714, and a precision of 0.833, while maintaining balanced classification performance with a sensitivity of 0.625 and specificity of 0.960. The random forest model yielded a slightly lower but still strong AUC of 0.930, with similarly high performance across other metrics. The SVM model achieved a higher sensitivity (0.750), but its precision (0.600) and specificity (0.840) were inferior to those of XGBoost. The LR model showed relatively lower performance across all metrics. Overall, both the XGBoost and random forest models achieved excellent predictive capability, with XGBoost showing marginally superior overall performance.

**TABLE 1:** Performance comparison of four models

Model	AUC	Accuracy	F1 score	Precision	Sensitivity	Specificity
LR	0.840	0.758	0.600	0.500	0.750	0.76
RF	0.930	0.879	0.714	0.833	0.625	0.96
XGBoost	0.950	0.879	0.714	0.833	0.625	0.96
SVM	0.855	0.818	0.667	0.600	0.750	0.84

**Fig 1:**
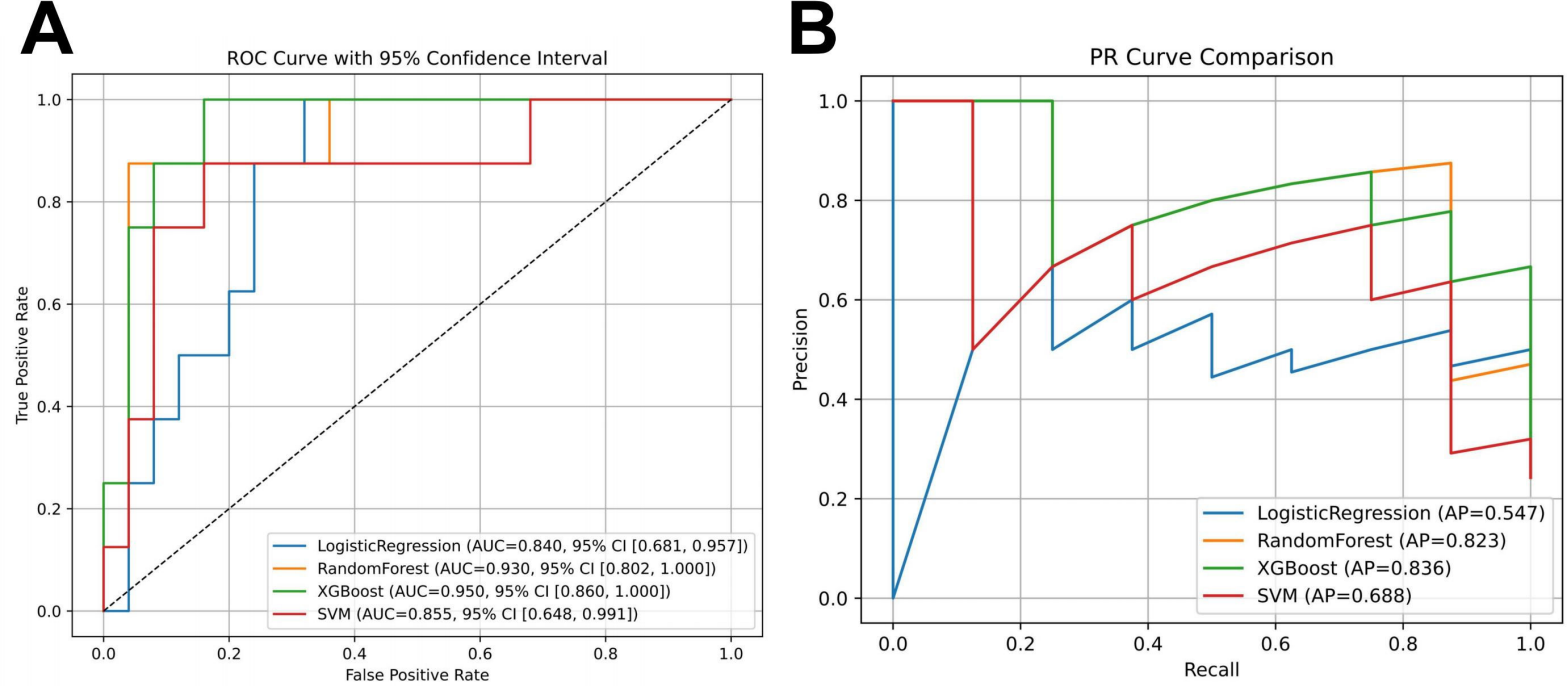
Evaluation of the discriminative performance of four ML models in mortality risk prediction for SFTS patients. (**A**) ROC curve analysis of four ML models. (**B**) PR curve analysis evaluating each model’s accuracy in identifying positive cases under imbalanced data conditions.

To further visualize and quantify the performance of each model across multiple dimensions, we generated a series of evaluation plots. Receiver operating characteristic (ROC) curve analysis revealed that XGBoost had the best overall discriminative ability (AUC = 0.950, 95% CI: 0.860–1.000), outperforming RF (AUC = 0.930, 95% CI: 0.802–1.000), SVM (AUC = 0.855, 95% CI: 0.648–0.991), and LR (AUC = 0.840, 95% CI: 0.681–0.957) (Fig. 1A). To verify the stability of performance estimates, we further evaluated the variation of AUC values for the XGBoost model across five-fold, three-times repeated cross-validation (CV). The results showed an average AUC of 0.950 ± 0.034 with a 95% CI of [0.882, 0.984], indicating minimal fluctuation among folds and demonstrating good model robustness and generalization potential. Further precision-recall (PR) curve analysis demonstrated that XGBoost also performed best under imbalanced data conditions, with an average precision (AP) of 0.836, slightly higher than RF (0.823), and significantly better than SVM (0.688) and LR (0.547) (Fig. 1B). These findings indicate that XGBoost excels at identifying mortality cases and effectively controls the false positive rate, achieving a superior balance between precision and recall.

The calibration curve of the model showed that XGBoost produced predicted probabilities that closely aligned with the observed event rates across most probability intervals, with a good fit along the diagonal line. This indicates high reliability in its predictions across different risk levels (Fig. S1A). Decision curve analysis (DCA) further confirmed its clinical applicability as the XGBoost model yielded consistently higher net benefits across a wide range of risk thresholds (0.1–0.6), outperforming other models as well as the extreme strategies of “treat-all” or “treat-none” (Fig. S1B). Confusion matrix analysis provided an intuitive view of classification performance. On the test set, the XGBoost model correctly classified 5 true positive (TP) mortality cases and 24 true negative (TN) survival cases, with 1 false positive (FP) and 3 false negative (FN) predictions, achieving a high specificity (0.96) and stable sensitivity (0.625). This reflects the model’s ability to detect positive cases while minimizing false alarms effectively. In contrast, although the SVM model achieved higher sensitivity (0.875), its specificity and accuracy were lower, resulting in overall inferior performance compared to that of XGBoost (Fig. S1C).

Taken together, both the XGBoost and random forest models demonstrated robust generalization performance under small-sample conditions. Among them, XGBoost showed a slight advantage in overall metrics and was, therefore, selected as the primary model for subsequent risk prediction and interpretability analyses.

### Discriminative and fitting performance evaluation of the Boruta-XGBoost model

To further enhance model performance and the robustness of feature selection, the Boruta algorithm was applied to all input variables using multiple resampling iterations and comparisons with shadow features, yielding 20 robust features (Fig. S2A). These features encompassed inflammatory cytokines, metabolic and hepatic–renal function indicators, and immune cell ratios, collectively reflecting the patients’ systemic inflammatory and immunometabolic status. To determine the optimal balance between model complexity and performance, features identified by Boruta were sequentially added according to their importance (*K* = 1–20), and model performance was evaluated using five-fold, three-times repeated cross-validation (5 × 3 repeated CV). The results showed that the AUC increased rapidly with additional features and reached a plateau when *K* ≈ 15. Based on the 1-SE rule, the optimal number of features was determined to be *K* = 15* (Fig. S2B). Notably, although comparable discriminative performance could be achieved with a smaller number of features (e.g., *K* ≈ 7–8), the AUC exhibited relatively larger variability across cross-validation folds within this range. In contrast, the feature subset corresponding to *K* = 15* maintained similar discriminative ability while demonstrating a more stable performance distribution and was, therefore, selected for subsequent model development and analyses. These findings indicate that the 15 selected features were sufficient to support the model’s predictive capability, achieving an optimal balance between performance and complexity and providing a foundation for the subsequent construction of a simplified model.

After determining the optimal number of features (K* = 15), a simplified XGBoost model was constructed based on this feature set and subsequently evaluated for performance. A summary of performance metrics is presented in Table 2. The results showed that the optimized Boruta-XGBoost model achieved comparable overall performance to the full-feature model, with both AUC and accuracy reaching 0.950 and 0.879, respectively, indicating that the simplification did not compromise the model’s discriminative capability. Notably, the simplified model demonstrated superior performance in F1 score (0.750 vs. 0.714) and sensitivity (0.750 vs. 0.625), suggesting improved ability to identify positive cases and better classification balance. Although there was a slight decrease in precision (0.750 vs. 0.833) and specificity (0.920 vs. 0.960), the higher sensitivity is of greater clinical relevance in the context of mortality risk prediction, where minimizing missed diagnoses is a priority. In summary, the optimized Boruta-XGBoost model achieved enhanced balance and predictive power while significantly reducing the number of input features, offering strong support for subsequent model deployment and clinical application.

**TABLE 2:** Performance comparison between XGBoost and Boruta-XGBoost^*a*^

Model	AUC (95% CI)	Accuracy	F1 score	Precision	Sensitivity (95% CI)	Specificity
XGBoost	0.950 (0.860–1.000)	0.879	0.714	0.833	0.625 (0.400–0.781)	0.960
Boruta-XGBoost	0.950 (0.900–0.977)	0.879	0.750	0.750	0.750 (0.521–0.887)	0.920

^*a*^ Note: AUC and sensitivity 95% confidence intervals were obtained using stratified bootstrap resampling on the fixed test set (B = 2,000).

A series of evaluation plots were generated on the test set to further visualize and quantify the performance of the Boruta-XGBoost model across different dimensions.

**Fig 2:**
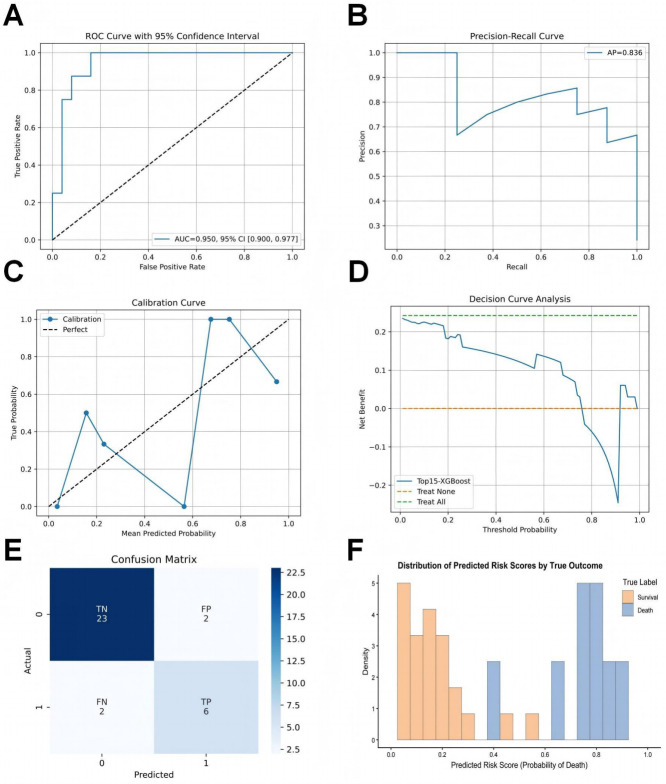
Performance evaluation of the Boruta-XGBoost model. (**A**) ROC curve; (**B**) PR curve; (**C**) calibration curve evaluating consistency between predicted probabilities and actual outcomes; (**D**) DCA assessing net clinical benefit across different threshold values; (**E**) confusion matrix showing the correspondence between true labels and predicted results; (**F**) distribution of predicted risk scores comparing survival and mortality groups.

The ROC curve demonstrated good discriminative ability, with an AUC of 0.950 (95% CI: 0.900–0.977) (Fig. 2A), indicating the model’s effectiveness in distinguishing between survival and mortality cases. Additionally, PR curve analysis showed robust performance in handling class imbalance, with an AP of 0.836 (Fig. 2B).

The calibration curve evaluated the consistency between predicted probabilities and observed event rates. The results showed that the model fitted well with the ideal diagonal line across most probability intervals, with deviations observed only in some high-risk segments (Fig. 2C), indicating good calibration performance. DCA further confirmed the model’s clinical utility, demonstrating a clear net benefit across a wide range of risk thresholds (0.1–0.75), outperforming both the “treat-all” and “treat-none” strategies (Fig. 2D). Evaluation of the confusion matrix showed that the model correctly identified 23 TN and 6 TP cases, with 2 FP and 2 FN in the test set. The model achieved a sensitivity of 0.750, a specificity of 0.920, and an overall accuracy of 0.879 (Fig. 2E), indicating a well-balanced performance in predicting mortality events. Considering all evaluation metrics, the optimized XGBoost model demonstrated robust predictive ability and potential clinical utility in the studied population.

We visualized the predicted mortality probabilities for all patients in the test set to assess the model’s discriminative ability between outcome groups. The results showed a clear separation in risk score distributions between the mortality and survival groups. Patients in the mortality group predominantly had predicted probabilities above 0.5, while those in the survival group were mainly distributed below 0.3 (Fig. 2F). These findings further validated the model’s strong discriminative power and were consistent with the earlier ROC and PR curve analyses. The evident separation in distribution also suggested that the model’s predicted probabilities possess clinical interpretability and practical utility, with promising potential to assist in identifying and intervening with high-risk patients in real-world clinical settings.

### Model interpretability analysis

**Fig 3:**
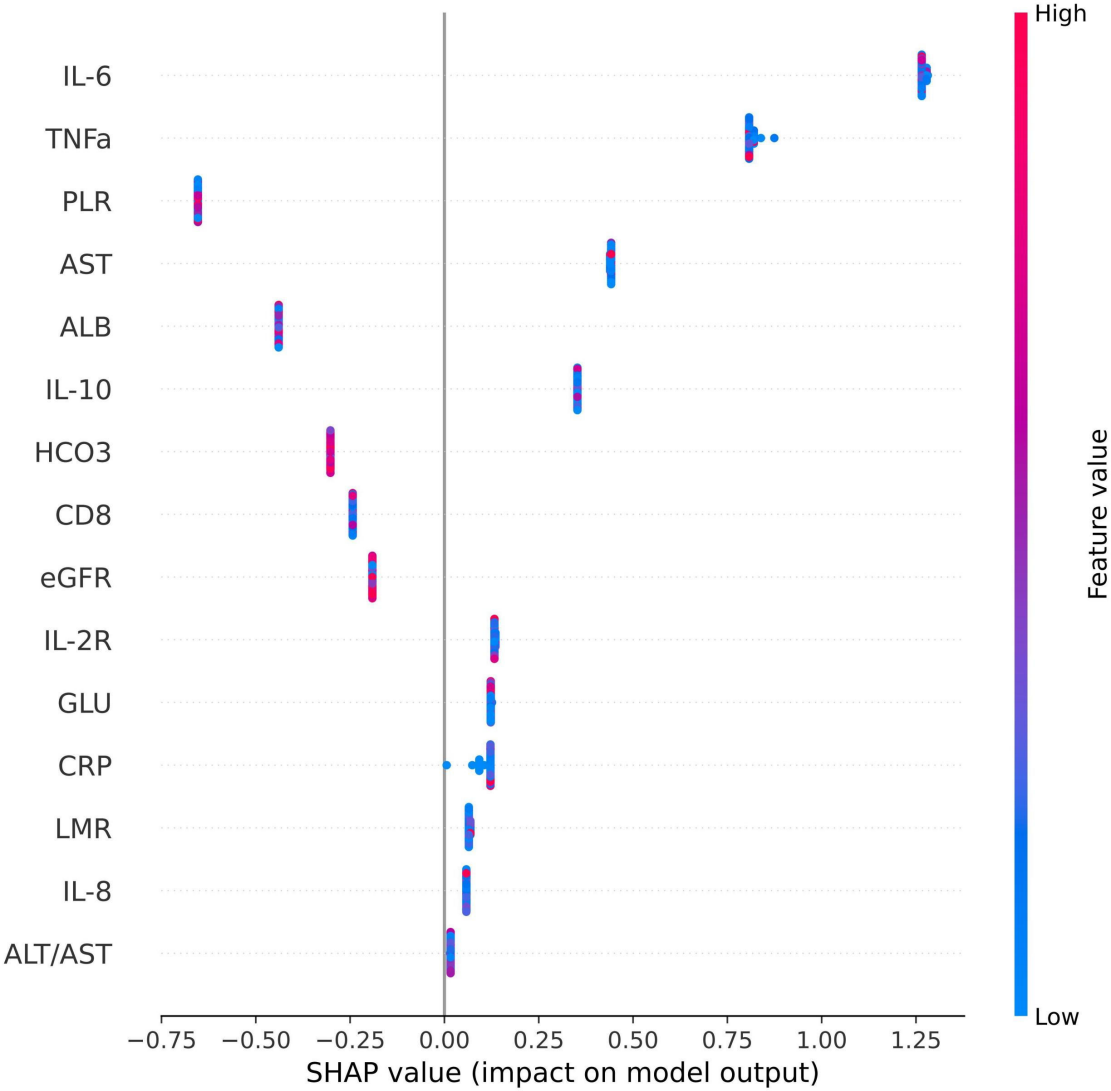
SHAP global explanation plot showing the feature contribution to mortality risk prediction. Red indicates higher feature values, while blue indicates lower values; the *X*-axis represents each feature’s positive or negative contribution to the prediction outcome.

We further applied the Shapley additive explanations (SHAP) algorithm to perform interpretability analysis on the optimized Boruta-XGBoost model. The results are shown in the figure. The SHAP summary plot illustrated the relative importance of each variable in predicting mortality risk. IL-6, TNF-α, PLR, AST, and ALB ranked as the top five contributing features. Specifically, the SHAP values for IL-6, TNF-α, and AST were predominantly positive, suggesting that elevated levels of these markers significantly increased mortality risk. In contrast, PLR and ALB showed negative contributions, indicating that higher levels might exert protective effects (Fig. 3). Notably, certain features, such as the PLR, exhibited relatively small variations in SHAP values across samples. This phenomenon does not indicate computational error but rather reflects the underlying feature distribution and the model’s learning patterns. First, the overall range of PLR values in this study population was narrow, leading to relatively stable marginal effects across individuals. Second, during the tree-splitting process, the Boruta-XGBoost model established relatively fixed partition thresholds for PLR, resulting in a consistent influence on the predicted probabilities. In addition, the SHAP algorithm averages feature contributions across all trees in an ensemble model, which tends to produce “near-constant” contribution values for features with low variance. Collectively, this pattern suggests that PLR exerts a stable and consistent protective effect in predicting SFTS mortality risk, rather than indicating model overfitting or interpretive bias.

Overall, the interpretability analysis indicates that the model primarily relies on indicators related to inflammatory response, metabolic function, and immune status when predicting mortality risk. These findings highlight the statistical links between these biological processes and disease severity, providing valuable insights for subsequent mechanistic investigations.

### Individual-level model interpretability (force plot analysis)

To further explore the predictive mechanisms of the XGBoost model at the individual level, we employed SHAP force plots to visualize explanations for representative mortality and survival cases. The results showed that, in one patient predicted as high risk for death, the model output a mortality probability of 0.691, markedly higher than the overall baseline probability of 0.277, indicating a clear model-estimated risk of death for this individual. In this case, variables such as AST, IL-6, and TNF-α contributed positively to the model output, whereas IL-10, CD8, CRP, and HCO₃⁻ exhibited suppressive effects (Fig. S3A).

Conversely, in a representative patient predicted to survive (low-risk group), the model output a mortality probability of only 0.107, substantially lower than the same baseline of 0.277. Here, HCO_3_^−^ emerged as the strongest protective feature, exerting the greatest positive contribution toward survival, while AST, IL-6, and TNF-α notably decreased the model’s death probability, shifting the overall prediction toward survival (Fig. S3B).

Taken together, these findings demonstrate that the XGBoost model integrates multidimensional biological information—including inflammatory responses, immune regulation, and metabolic function—to achieve individualized differentiation of mortality risk, underscoring its precision and interpretive capacity for personalized clinical prognostic assessment.

In summary, through systematic feature selection and hyperparameter optimization, we developed an XGBoost-based mortality risk prediction model built upon 15 key variables identified by the Boruta algorithm. This model maintained the full-feature version’s discriminative power and fitting performance while significantly reducing the feature dimension. It demonstrated improved sensitivity and classification balance, indicating strong clinical practicality and potential for broader application. Performance metrics such as AUC, accuracy, sensitivity, and specificity remained high in the test set. Moreover, calibration and decision curves validated the model’s predicted probabilities, confirming their reliability and clinical net benefit. SHAP-based interpretability analysis further revealed that IL-6, TNF-α, PLR, and AST played critical roles in driving the model’s output, enhancing its transparency and explainability. At the individual level, the model demonstrated its ability to integrate multidimensional data—including inflammatory, immune, and metabolic markers—to accurately identify high-risk patients, offering theoretical and technical support for future deployment and clinical translation.

### MR analysis of metabolic and inflammatory factors

To identify key metabolic, inflammatory, and immune-related indicators and to explore their synergistic interaction patterns, we first conducted differential analyses of the 15 key variables selected by the Boruta algorithm between the death and survival groups. The results showed that ALT/AST, eGFR, AST, IL-10, IL-2R, IL-6, IL-8, TNF-α, and CD8 differed significantly between the two groups (*P <* 0.05) (Fig. S4A). Subsequent Spearman correlation analysis revealed significant associations between several metabolic indicators (e.g., AST, eGFR, and GLU) and inflammatory markers (e.g., IL-6, IL-10, and IL-8) as well as the immune factor CD8 (Fig. S4B), thereby providing data support for constructing metabolic–inflammatory–immune tri-nodal pathways.

To further investigate potential causal relationships among metabolic, inflammatory, and immune factors, we conducted Spearman correlation analyses based on the 15 key variables identified by the Boruta algorithm and subsequently constructed a metabolism–inflammation–immunity tri-nodal pathway. Pairwise relationships with absolute correlation values greater than 0.3 and statistically significant *P*-values were selected. Six representative pathways were ultimately identified: AST → IL-6 → CD8, AST → IL-10 → CD8, AST → IL-8 → CD8, GLU → IL-10 → CD8, eGFR → IL-10 → CD8, and eGFR → IL-8 → CD8. These pathways were subsequently subjected to validation using a two-step MR approach.

**Fig 4:**
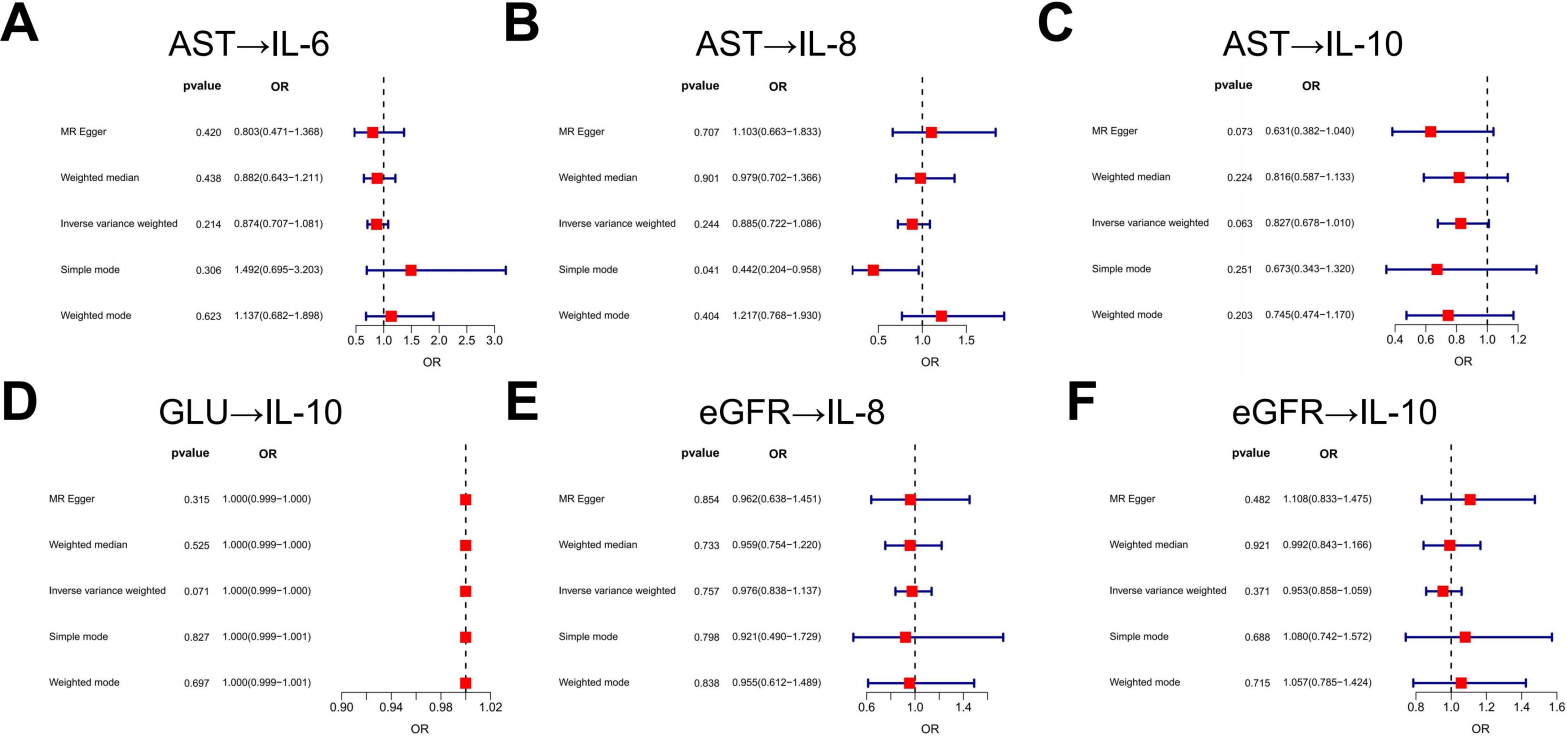
MR results of metabolic factors and inflammatory markers. (**A–F**) Forest plots of MR results for AST→IL-6 (**A**), AST→IL-8 (**B**), AST→IL-10 (**C**), GLU→IL-10 (**D**), eGFR→IL-8 (**E**), and eGFR→IL-10 (**F**).

In this study, we evaluated six potential causal pathways from metabolic factors to inflammatory factors, including AST → IL-6, AST → IL-8, AST → IL-10, GLU → IL-10, eGFR → IL-8, and eGFR → IL-10. Results from multiple MR methods showed that only the AST → IL-8 pathway yielded statistical significance using the simple mode method (odds ratio [OR] = 0.442, 95% CI: 0.204–0.958, *P =* 0.041). For all other pathways, *P*-values from all MR methods were greater than 0.05, the ORs were generally close to 1, and CIs crossed the null line, indicating no consistent or robust causal effects were observed (Fig. 4A through F).

Scatter plot results demonstrated consistent regression line directions across all five MR methods in the AST → IL-6, AST → IL-10, GLU → IL-10, and eGFR → IL-10 pathways, with slopes generally approaching zero, suggesting strong consistency among different methods. In the AST → IL-8 and eGFR → IL-8 pathways, slight deviations in the direction of fitted lines were observed for some methods, but no systematic bias was detected in overall trends (Fig. S5A through F).

Single-nucleotide polymorphism (SNP)-level forest plots showed that the effect estimates for individual instrumental variables were evenly distributed. The combined effect directions from MR Egger and inverse variance weighted (IVW) models were consistent, indicating that no single SNP exerted a dominant influence on the causal estimates (Fig. S6A through F). Leave-one-out sensitivity analysis further assessed the influence of key SNPs on result stability. In all six pathways, removing any single SNP did not significantly alter the effect estimates, demonstrating the robustness and reliability of the findings (Fig. S7A through F).

In heterogeneity testing, *P*-values for Cochran’s Q tests were all greater than 0.05 (Table S1), indicating no significant heterogeneity among the selected instrumental variables. Furthermore, intercept tests from MR Egger showed no significant deviation (*P >* 0.05 for all), ruling out the presence of horizontal pleiotropy (Table S2).

The current MR analyses did not provide consistent or robust statistical evidence to support causal pathways from metabolic to inflammatory factors.

### MR analysis of inflammatory and immune factors

**Fig 5:**
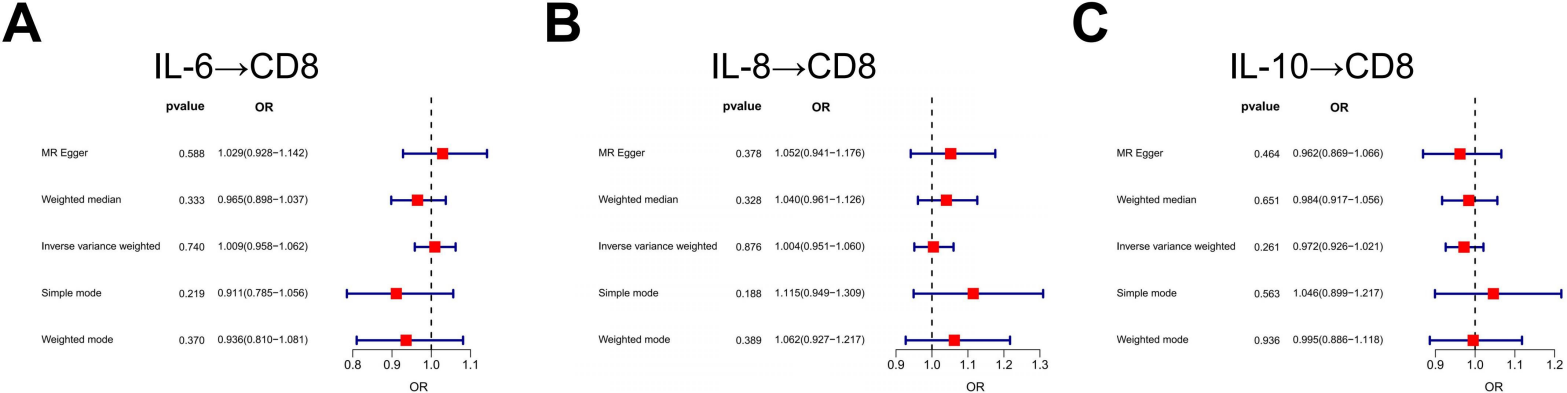
MR results for inflammatory and immune factors. (**A–C**) Forest plots of MR results for IL-6→CD8 (**A**), IL-8→CD8 (**B**), and IL-10→CD8 (**C**).

To further investigate the causal effect of inflammatory factors on the immune factor CD8^+^ T cell levels, we included IL-6, IL-8, and IL-10 as exposure variables and constructed three corresponding causal pathways: IL-6 → CD8, IL-8 → CD8, and IL-10 → CD8. The results showed that, across all pathways, the ORs obtained from the main MR methods (IVW, MR Egger, weighted median, simple mode, and weighted mode) were all close to 1. Their 95% CIs crossed the null line, and all *P*-values were greater than 0.05, indicating no statistical significance (Fig. 5A through C).

The scatter plots showed that the regression lines fitted by the five MR methods for each group had similar directions, with no obvious divergence, suggesting high consistency among the methods (Fig. S8A through C). The SNP-level forest plots further confirmed that the effect estimates of the instrumental variables were evenly distributed, and the combined estimates from the MR Egger and IVW models were directionally consistent (Fig. S9A through C). Sensitivity analysis using the leave-one-out method indicated that no single SNP dominated the overall effect estimate, supporting the robustness of the model (Fig. S10A through C).

In the heterogeneity test, the IL-6 → CD8 and IL-8 → CD8 pathways showed some degree of heterogeneity, with Q-test *P*-values of 0.017 and 0.061, respectively, while no significant heterogeneity was observed in the other pathways (Table S1). In the pleiotropy analysis, the Egger intercept *P*-values for all pathways were greater than 0.05, ruling out substantial horizontal pleiotropy (Table S2). The current MR analysis did not provide sufficient statistical evidence to support a stable causal relationship between inflammatory and immune factors.

Although a series of systematic MR analyses were conducted based on multiple metabolism-inflammation-immunity pathways, no consistent and robust statistical evidence was found to support clear causal relationships among these factors.

## DISCUSSION

SFTS is a highly fatal infectious disease caused by the SFTS virus, which has been widely prevalent in East Asia in recent years (30). Due to its complex clinical manifestations and rapid disease progression, accurate prognosis prediction holds significant clinical value (7). Most current studies focus on the association between individual biochemical indicators or clinical features and risk, lacking systematic integration and causal inference approaches. This limitation hinders the development of precision medicine strategies (31–33). This study integrated ML and MR to advance SFTS research from two dimensions: mortality risk prediction and mechanistic exploration, providing a technically feasible path with greater translational potential. This dual-track strategy of “prediction + interpretation” not only enhances the practical utility of the model but also promotes the shift in SFTS mechanism research from descriptive analysis to causal reasoning, demonstrating both foresight and innovation.

Regarding predictive modeling, we systematically compared the performance of four mainstream algorithms and identified XGBoost as the best-performing method for mortality risk prediction (AUC = 0.950; accuracy = 0.879). Compared with traditional LR, XGBoost showed superior ability in handling complex interactions and nonlinear relationships among variables. Unlike traditional models that assume linear relationships among variables, machine learning approaches—such as random forest, support vector machine, and XGBoost—can flexibly capture complex nonlinear relationships and interactions among laboratory and immunological indicators. This capability is particularly important in the study of SFTS as disease progression involves multilevel coupling among metabolic, inflammatory, and immune processes that cannot be adequately characterized by simple linear models. The constructed Boruta-XGBoost model maintained high predictive performance while significantly reducing variable count (with the F1 score improved to 0.750), enhancing clinical applicability. This model demonstrated stronger generalizability and prediction stability than previous methods relying on single-indicator evaluation. These findings suggest that the introduction of ensemble learning models in infectious disease research can effectively address challenges such as variable complexity and limited sample sizes, providing a valuable reference for future modeling in related diseases.

In addition to predictive performance, we emphasized model interpretability by applying the SHAP method to identify the impact of individual variables on model output. IL-6, TNF-α, PLR, and AST had the highest weights in mortality prediction, consistent with previously reported inflammatory and organ dysfunction markers. Notably, PLR, an inflammation-related ratio, although not widely reported in SFTS studies (34), demonstrated strong explanatory power in our model, indicating its potential clinical relevance. Ranking and interpreting variable importance helps enhance clinicians’ trust in the model output and provides quantitative support for further investigation into disease mechanisms. Although some variables (e.g., PLR) exhibited limited variation in SHAP values across individuals, this primarily reflects the stable distribution of the feature within the overall sample and the fixed threshold learned by the model for that variable, rather than overfitting or interpretive bias. Such patterns are common in small-sample settings or when features show low variance, suggesting that future studies could enhance the dynamic interpretability of the model by expanding the sample size or incorporating higher-resolution variable inputs.

This study also innovatively incorporated MR to explore potential causal pathways among metabolic, inflammatory, and immune factors. Using genetic variants as instrumental variables helps overcome confounding and reverse causality issues, thereby addressing limitations inherent in traditional correlation-based analyses. Six variable pathways were selected through Spearman correlation analysis and subsequently tested for causality using MR methods, marking an important step forward in mechanistic exploration. Although most pathways did not reach statistical significance, the AST → IL-8 pathway exhibited significant causal associations, suggesting that metabolic function may influence disease progression through inflammatory mediation. To our knowledge, this is the first report of such findings in the context of infectious disease, highlighting their theoretical innovation.

The AST → IL-8 pathway implies that hepatic dysfunction may exacerbate SFTS severity via inflammatory pathways, while the positive association in the eGFR → IL-10 pathway may reflect the influence of renal function changes on anti-inflammatory responses. Although causal analyses of IL-6, IL-8, and IL-10 on the immune marker CD8 did not reach significance, the observed trends still suggest potential biological relevance. While these preliminary findings are insufficient to establish a complete causal network, they offer testable directions for future studies—especially with the integration of multi-omics data or expanded sample sizes, which may uncover more stable mechanistic pathways.

This study also conducted sensitivity analysis, heterogeneity testing, and pleiotropy evaluation alongside MR analysis to ensure the robustness of conclusions. The use of multiple estimation methods to cross-validate causal relationships enhanced the reliability of inference. Compared to traditional single-model analyses, this rigorous approach is better suited to address the multifactorial nature of infectious diseases. Particularly for emerging diseases such as SFTS, the MR framework provides a novel paradigm for mechanism-oriented research and may be extended to causal investigations in other infectious diseases.

Most existing SFTS-related studies have focused on a single dimension (e.g., inflammatory factors and viral load) (35, 36), whereas this study integrated variables across metabolic, inflammatory, and immune dimensions to construct a more comprehensive analytical framework. This study improved accuracy and applicability in terms of prediction compared to traditional regression models. In terms of mechanism, the introduction of MR filled the gap in causal inference, enhancing the depth and scientific value of the research. Although a complete and stable three-node causal pathway was not identified, several key causal relationships were preliminarily captured, offering clear directions for future multi-omics integration and new insights into the etiology of SFTS.

From a clinical perspective, the Boruta-XGBoost model developed in this study enables quantitative assessment of mortality risk during the early stage of hospital admission. The model’s output probability can serve as a clinical decision-support tool: when the predicted probability exceeds 0.5, it indicates a potentially high risk of death, warranting intensified monitoring and timely interventions—such as early initiation of renal protection, management of hepatic injury, and control of excessive inflammatory responses. For patients with intermediate risk scores (0.3–0.5), dynamic reassessment based on changes in key variables such as IL-6, TNF-α, AST, and PLR is recommended. Moreover, the model-identified key features provide valuable insights into potential therapeutic targets. Elevated IL-6 and TNF-α levels highlight the central role of cytokine storms, suggesting that anti-inflammatory strategies, such as IL-6R antagonists or TNF-α inhibitors, may hold clinical research potential. The associations of AST and eGFR with mortality emphasize the importance of liver and kidney protection, indicating that early organ-supportive therapy may improve outcomes. Future research integrating longitudinal monitoring and multi-omics analyses may further enable individualized intervention strategies based on these biomarkers, ultimately achieving a closed-loop application linking model prediction with therapeutic decision-making.

Despite its methodological innovations, this study has several limitations. First, the sample size was relatively small, including only 107 patients, which may affect the stability of the model and the statistical power of causal inference. It should be noted that in small-sample settings, cross-validation may introduce optimistic bias in performance estimation. To mitigate this risk, model training and evaluation were conducted within a nested cross-validation framework, and the standard deviation of AUC values across folds was reported to reflect model stability. The results demonstrated minimal fluctuation in AUC across different data partitions, indicating that the performance estimates were relatively robust. Second, some potentially important variables, such as viral load and dynamic clinical indicators, were not included, possibly omitting valuable predictive information. In the MR analysis, the lack of well-established SFTS-specific genome-wide association study (GWAS) data limited the availability of suitable instrumental variables, potentially affecting the accuracy of causal inference. Moreover, the study was designed as a single-center retrospective analysis without external data set validation, limiting the generalizability of the findings. Therefore, the conclusions should be further validated in large-scale, multicenter prospective studies.

Future research should expand the sample size through multicenter collaboration and conduct prospective cohort studies to enhance the model’s generalizability. It is also recommended to incorporate dynamic clinical variables and longitudinal data, employing time-series analysis to capture disease progression better. For mechanistic exploration, integrating multi-omics data (e.g., transcriptomics, proteomics, and metabolomics) may help construct a complete metabolic–inflammatory–immune causal network, deepening the understanding of the systemic pathophysiology of SFTS. Furthermore, efforts should be made to develop comprehensive genetic databases related to SFTS, providing higher-quality instrumental variables for MR. The ultimate goal is to translate these findings into intelligent clinical decision-making tools that enable early screening and intervention, thereby improving the survival rate of patients with SFTS.

### Conclusion

Based on clinical data, this study developed a mortality risk prediction model for SFTS patients, in which the XGBoost algorithm demonstrated superior discriminative ability (AUC = 0.950) and high efficiency in feature handling. The subsequently constructed Boruta-XGBoost model enhanced sensitivity while maintaining high accuracy, thus balancing predictive performance with clinical applicability. Key features such as IL-6, TNF-α, AST, and PLR are routinely measured indicators, supporting the model’s potential for practical deployment and early risk identification.

**Fig 6:**
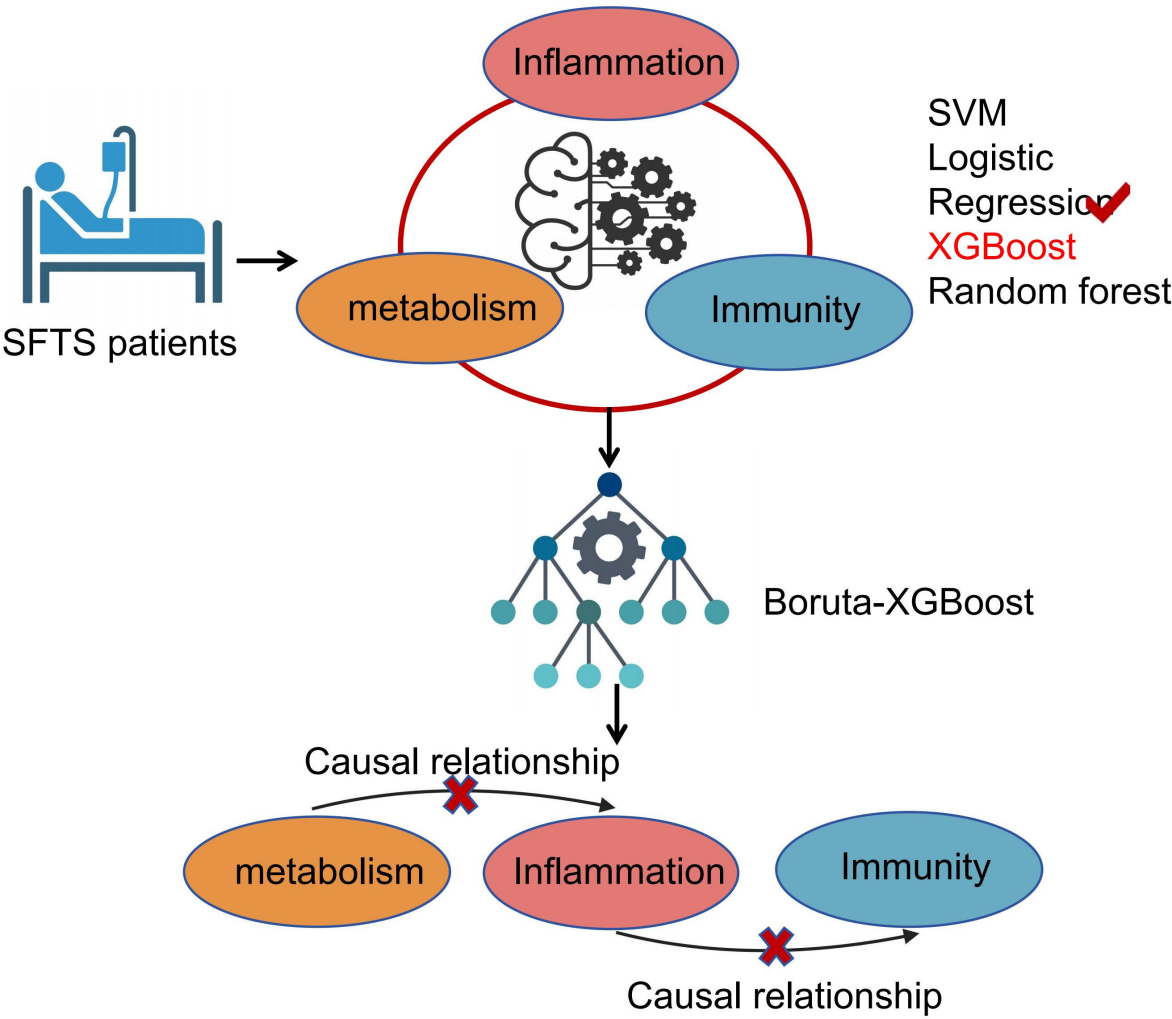
Workflow diagram of the metabolic–inflammatory–immune interaction network study based on ML and MR. Created in BioRender.

By integrating machine learning and Mendelian randomization methods, the study explored both predictive modeling and causal inference. Although most causal pathways did not reach statistical significance, the results for the AST → IL-8 and eGFR → IL-10 pathways suggested potential metabolic–inflammatory regulatory links, providing new directions for mechanistic research into SFTS (Fig. 6).

This study proposes an integrated strategy that combines ML model interpretability with causal inference to identify key biological mechanisms, offering a novel paradigm for precision prediction and mechanistic investigation of infectious diseases. The developed model demonstrates strong translational potential, with the ability to support early identification of high-risk patients and inform optimized resource allocation and intervention strategies in clinical practice.

## MATERIALS AND METHODS

### Computational environment and software versions

All analyses were conducted within a standardized computational environment. ML modeling and interpretability analyses were performed using Python 3.9.18, with the following key package versions: scikit-learn 1.3.1 (37), xgboost 1.7.6 (38), imbalanced-learn 0.11.0 (39), pandas 2.0.3, numpy 1.25.2, matplotlib 3.8.0, seaborn 0.12.2 (40), and scipy 1.11.x.

Mendelian randomization analyses were conducted in R 4.3.1, using the following main packages: TwoSampleMR 0.5.7, MRPRESSO 1.0, gwasglue 0.4.2, and ggplot2 3.4.4.

### Data sources and study subjects

This study retrospectively collected clinical data from patients diagnosed with SFTS at The First Affiliated Hospital of Anhui Medical University. The data set included various laboratory indicators such as inflammatory cytokines, liver and kidney function parameters, electrolytes, and blood cell ratios. The discharge outcome (mortality/survival) was used as the target variable for prediction, and a binary classification model was constructed accordingly. All data were de-identified and complied with ethical review requirements (Ethical Approval Number: Quick-PJ2021-06-18). All laboratory test results were obtained from the first examination conducted at hospital admission, defined as measurements completed within 24 h of confirmed diagnosis. For patients with multiple test records within the first day of admission, only the earliest result was used as model input. This design ensured that the model relied on information available at an early clinical stage for risk prediction, thereby minimizing the potential impact of dynamic changes in biomarkers during disease progression on model stability. It should be noted that although the sampling time was uniformly restricted to within 24 h after diagnosis, the severity of disease at the time of presentation and diagnosis may have varied among patients. In some cases, patients may have already been at a relatively advanced stage of disease upon hospital admission. Therefore, the model developed in this study is positioned as a risk stratification tool based on laboratory indicators that are available at the early stage of admission/diagnosis. Using the earliest available test results within this unified time window as model inputs remains clinically reasonable and operationally feasible.

### Data preprocessing and feature engineering

Missing values in continuous variables were imputed using the median. The label variable “survival status” was converted into a binary variable (mortality = 1; survival = 0). The SMOTE method was applied for oversampling during model training to address class imbalance. N features were included and standardized using StandardScaler before being fed into the modeling pipeline.

### Model construction and comparative evaluation

The data set comprised 107 patient samples, which were randomly divided into a training set (*n* = 75) and an independent test set (*n* = 32) in a 70:30 ratio. Stratified sampling based on the outcome variable (survival/death) was employed to maintain balanced class proportions between the two subsets. A total of 27 initial features were included in the modeling process after median imputation for missing values, one-hot encoding of categorical variables, and feature standardization. All features were de-identified, and to ensure reproducibility of the study, the corresponding matrix of the 27 original features has been provided in a de-identified format in the supplemental material (Data S1).

This study employed four commonly used ML algorithms—LR, RF, SVM, and XGBoost—to develop predictive models for mortality risk in SFTS patients. All models were trained using a pipeline integrating SMOTE oversampling and feature standardization to handle class imbalance and ensure feature scale consistency. During training, repeated stratified *K*-fold cross-validation (*n*_splits = 5; *n*_repeats = 3) was used to evaluate model performance. GridSearchCV was applied to optimize hyperparameters for each model, using the area under the receiver operating characteristic (ROC) curve (AUC) as the scoring function.

The main parameters of each model were optimized using a grid search (GridSearchCV) under a fivefold, three-repeated cross-validation framework. The search ranges and final optimal parameters are summarized in Table S3. For the logistic regression model, the maximum number of iterations was set to 1,000, and the L2 regularization penalty term C was tuned. For the random forest, XGBoost, and support vector machine models, key hyperparameters, such as the number of base learners, tree depth, learning rate, kernel type, and kernel-specific parameters, were systematically tuned to achieve optimal performance.

Prediction results on the test set were output as predicted probabilities (predict_proba), from which final class labels were determined using a threshold of 0.5. All machine learning models produced standardized probability scores, where the predicted probability for the “death” class was defined as the individual risk score, representing the estimated probability (range: 0–1) of death for each patient. Predictions with a probability ≥ 0.5 were classified as high risk (death), whereas those with <0.5 were classified as low risk (survival). In cases where multiple samples yielded identical predicted probabilities, final class assignments were determined according to the algorithm’s internal node-splitting priority (e.g., in XGBoost, based on the order of split gain within the decision tree structure).

### Feature selection

To obtain a robust and interpretable feature set, feature selection was performed within a nested cross-validation (nested CV) framework. In each outer training fold, the Boruta algorithm (base learner: random forest) was first applied, using iteratively permuted shadow features until convergence, to identify “confirmed” important features. Subsequently, stability selection was conducted by performing 100 iterations of stratified resampling on the same training fold, recording the frequency with which each feature was selected by Boruta. A selection frequency ≥60% was used as the stability threshold. The final candidate feature set was defined as the intersection of features identified as “confirmed” by Boruta and those meeting the stability threshold.

Within the candidate set, an inner cross-validation procedure was used to construct a performance–feature number curve (performance vs. *K*), in which variables were sequentially added according to their ranked feature importance. The optimal number of features (K*) was determined based on the 1-standard error (1-SE) criterion, whereby a more parsimonious model was selected under the condition that the mean AUC had reached a performance plateau and showed no statistically significant difference from the maximum achievable performance. It should be noted that although comparable discriminative performance could already be achieved with a smaller number of features (e.g., *K* ≈ 7–8), a final value of *K* = 15* was selected to balance model robustness and stability across different data partitions, while retaining more comprehensive clinical and biological information. This choice also facilitates subsequent model interpretability analyses and supports the exploration of metabolism-inflammation-immunity pathways.

### Boruta-XGBoost model construction

To enhance model robustness and clinical applicability, a Boruta-XGBoost predictive model was developed within the XGBoost algorithm framework. Model inputs consisted of the K* features identified through Boruta screening and performance-based optimization. The SMOTE method was applied to the training set to oversample the minority class and address class imbalance. To minimize bias in performance estimation under a small-sample setting, model construction was conducted within a nested cross-validation framework. The outer layer employed fivefold, three-times repeated stratified cross-validation (5 × 3 repeated stratified CV) for performance evaluation, while the inner layer was used for hyperparameter tuning and optimization, thereby preventing information leakage between training and validation data. During each outer iteration, the mean and standard deviation of the AUC were calculated to assess model stability across different data partitions. After standardizing the input features, model training and hyperparameter tuning were performed using repeated stratified *K*-fold cross-validation (five folds, three repeats). RandomizedSearchCV was used for hyperparameter optimization, performing 50 random searches within a broad parameter space. The parameters tuned included the following: learning_rate, *n*_estimators, max_depth, colsample_bytree, subsample, gamma, and scale_pos_weight.

After optimization, model performance was evaluated on an independent test set. Performance metrics included AUC, accuracy, F1-score, sensitivity, specificity, and precision, and the results were compared with the full-feature model and the Boruta-XGBoost model.

### Model evaluation and visualization

Model performance on the test set was evaluated using multiple metrics, including AUC, accuracy, F1-score, sensitivity, specificity, and precision. Visualization tools included the ROC curve, precision-recall (PR) curve, calibration curve, DCA, and a confusion matrix heatmap. The 95% confidence intervals (CIs) for AUC values were calculated using the bootstrap method. Confusion matrix analyses were based on predicted probabilities from the XGBoost model, with a fixed classification threshold of 0.5; samples with predicted probabilities ≥0.5 were classified as deaths (high risk), and those with <0.5 as survivors (low risk).

To quantify uncertainty in performance estimates, stratified bootstrap resampling (B = 2,000) was applied to the fixed test set. Positive and negative samples were resampled with replacement to preserve the original class ratio. For each resample, the ROC curve (AUC) and sensitivity were calculated, and 95% CIs were constructed using the median and 2.5th–97.5th percentiles of the bootstrap distributions. Other performance metrics, including accuracy, specificity, precision, and F1-score, were reported as point estimates.

### Model interpretability analysis

Model interpretability was assessed using the Shapley additive explanations (SHAP) framework. A SHAP summary plot was generated to identify the key variables most strongly influencing the prediction of mortality risk. In addition, SHAP waterfall plots were constructed for representative individual cases (deceased and surviving patients) to visualize model decision pathways, illustrating the positive or negative contributions of each feature to the model output and the overall direction of prediction.

### Comparison and correlation analysis of 15 robust features selected by Boruta

To further investigate the key variables associated with mortality outcomes, group comparisons and Spearman correlation analyses were performed based on the 15 robust features identified through Boruta selection. Between-group differences were assessed using the Mann–Whitney *U* test, with significance levels indicated by *P*-values. All visualizations were generated using the Xiantao Academic Platform (https://www.xiantaozi.com/).

#### Data collection and single nucleotide polymorphism (SNP) selection

The GWAS data related to the metabolic factors (AST, eGFR, and GLU), inflammatory factors (IL-6, IL-8, and IL-10), and immune factor (CD8) used in this study were obtained from the IEU OpenGWAS database (https://gwas.mrcieu.ac.uk/). Specifically, the data sets included the following: AST from bbj-a-8, eGFR from ieu-a-1284, and GLU from met-d-Glucose. For inflammatory markers, IL-6 was sourced from prot-a-1538 and IL-8 from prot-a-749. To minimize pleiotropy and improve the strength of instrumental variables, two data sets—prot-a-1464 and ebi-a-GCST004444—were used for IL-10. In most MR analyses involving IL-10, prot-a-1464 was employed. However, for the eGFR → IL-10 pathway, due to observed pleiotropy, the ebi-a-GCST004444 data set was used instead as it provided greater genetic explanatory power for IL-10 expression and showed no evidence of pleiotropic interference, thereby enhancing model robustness. All data sets used were publicly available and did not require additional ethical approval.

#### SNP selection

First, association analyses of exposure data sets were conducted using the “gwasglue” package in R. A *P*-value threshold of 5e-05 was applied to select SNPs strongly associated with the exposure traits to serve as instrumental variables. Next, the “TwoSampleMR” package in R was used to remove SNPs exhibiting linkage disequilibrium (LD), with filtering parameters set to kb = 10,000 and *r*² = 0.001, meaning SNPs within 10,000 kb and *r*² > 0.001 from the lead SNP were excluded. Subsequently, SNPs were filtered based on an *F*-statistic greater than 10 to minimize bias caused by weak instruments. The *F*-statistic was calculated using the following formula:


$$F=\frac{R^{2}}{1-R^{2}}\times \frac{n-k-1}{k}$$

Here, *n* represents the sample size of the GWAS data for the exposure variable, *k* refers to the number of SNPs selected as instrumental variables, and *R*² denotes the proportion of variance in the exposure explained by the k instrumental variables. The calculation formula is as follows:


$$R^{2}=\sum_{1}^{k}\left(\beta^{2}\times \left(1-EAF\right)\times 2EAF\right)$$

In the formula, β represents the effect size of the SNP used as an instrumental variable, and EAF refers to the effect allele frequency of the SNP. When *k* = 1, the statistical power of a single SNP can be calculated.

Finally, the PhenoScanner database was used to query the associations between the selected SNPs and phenotypes and to identify additional variables or factors related to the outcome variable that might act as confounders. Based on relevant literature, SNPs associated with potential confounding factors were excluded.

### MR analysis

Mendelian randomization–pleiotropy residual sum and outlier (MR-PRESSO) was used to obtain relatively stable causal effect estimates in the presence of outliers. Biased SNPs were identified and removed using MR-PRESSO. Subsequently, MR analyses were performed with the “TwoSampleMR” package in R, based on the filtered SNPs, using five methods: MR-Egger regression, weighted median, inverse variance weighted (IVW), simple mode, and weighted mode.

#### Heterogeneity test

Heterogeneity was assessed using Cochran’s *Q* test, which evaluates the variation in MR effect estimates. A *Q*-value less than 0.05 indicated the presence of heterogeneity; conversely, a *Q*-value greater than 0.05 indicated no significant heterogeneity.

#### Pleiotropy test

A *P*-value >0.05 suggested the absence of pleiotropy, while a *P*-value <0.05 indicated the presence of pleiotropy, implying that the instrumental variables might influence the outcome through pathways other than exposure.

#### Leave-one-out sensitivity analysis

This analysis was conducted by sequentially removing one SNP at a time and recalculating the combined effect estimate using the remaining SNPs. If the results remained consistent (i.e., all estimates aligned on one side of the vertical reference line), it indicated that no single SNP disproportionately influenced the causal association between the exposure and the outcome. This further demonstrated the robustness and reliability of the MR results in this study.

## Data Availability

The clinical data of the 107 patients with SFTS used in this study were rigorously anonymized and de-identified. Due to ethical considerations and privacy protection requirements, these data are not publicly available. All analysis scripts used in this study, including data preprocessing, feature selection, model training, performance evaluation, SHAP-based interpretability analyses, and Mendelian randomization analyses, have been deposited in a dedicated GitHub repository: https://github.com/bio-ai-Academic/SFTS_ML_MR. The repository provides the complete source code as well as representative example data sets sufficient to reproduce the full analytical workflow and the main results of this study. All data generated or analyzed during this study are included in this article and/or its supplemental files. Further inquiries can be directed to the corresponding author.

## SUPPLEMENTAL MATERIAL

Data S1 msphere.00831-25-s0001.xlsx Clinical data set used for machine learning analysis of mortality risk in patients with SFTS.

Supplemental material msphere.00831-25-s0002.docx Supplemental figures and tables.

ASM does not own the copyrights to Supplemental Material that may be linked to, or accessed through, an article. The authors have granted ASM a non-exclusive, world-wide license to publish the Supplemental Material files. Please contact the corresponding author directly for reuse.
