# Supplementary material for: Integrative analysis of mortality risk in SFTS using machine learning and genetic approaches
Source: mSphere. 2026 Jun 16;11(7):e00831-25. doi: 10.1128/msphere.00831-25 (PMC13410981; doi:10.1128/msphere.00831-25)
Supplement: Supplemental material — Supplemental figures and tables. [file msphere.00831-25-s0002.docx]

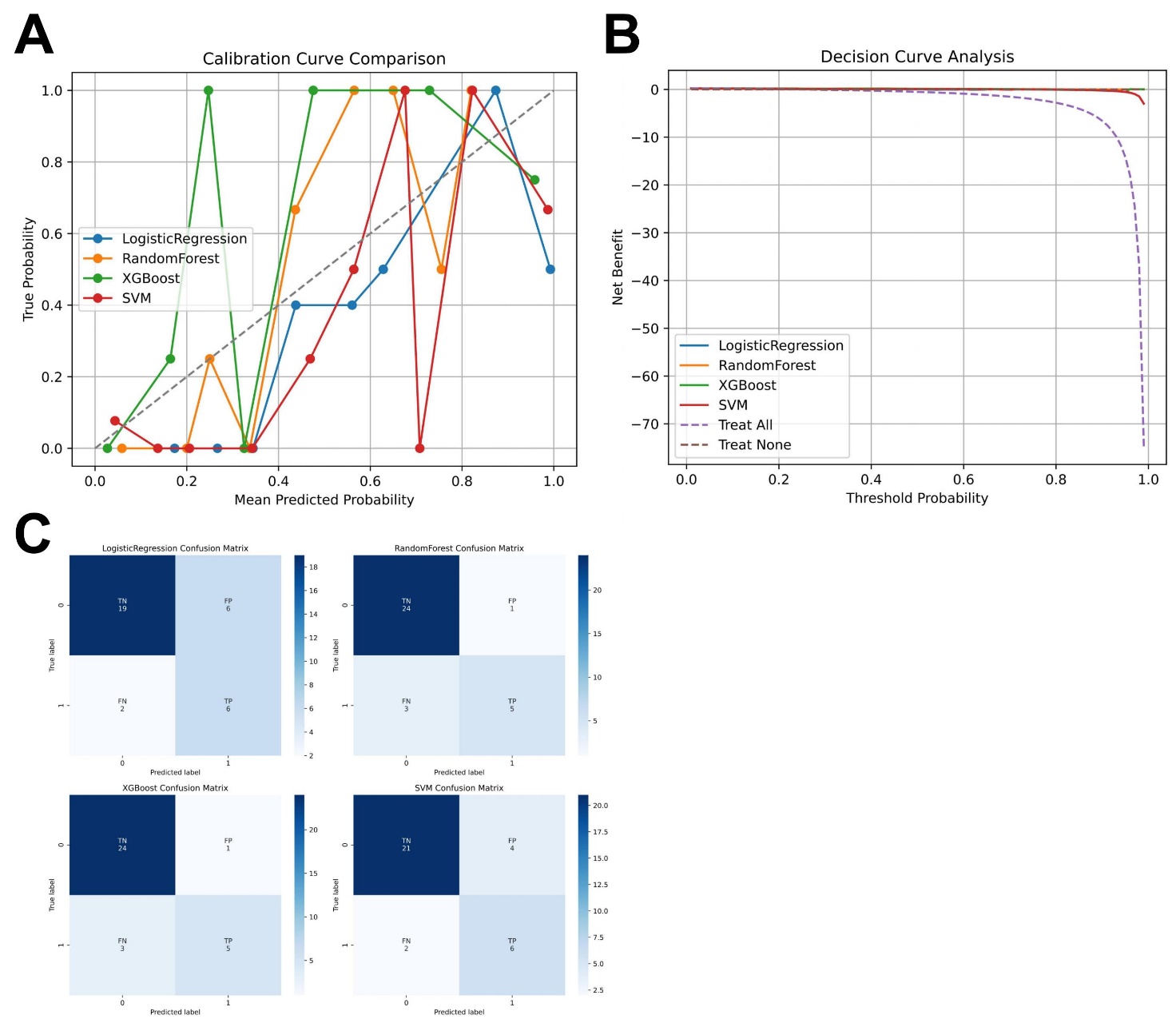


**Figure S1. Performance of four ML models across different evaluation dimensions in SFTS mortality risk prediction.** (A) Calibration curves of four ML models; (B) DCA; (C) Confusion matrix analysis of four ML models.


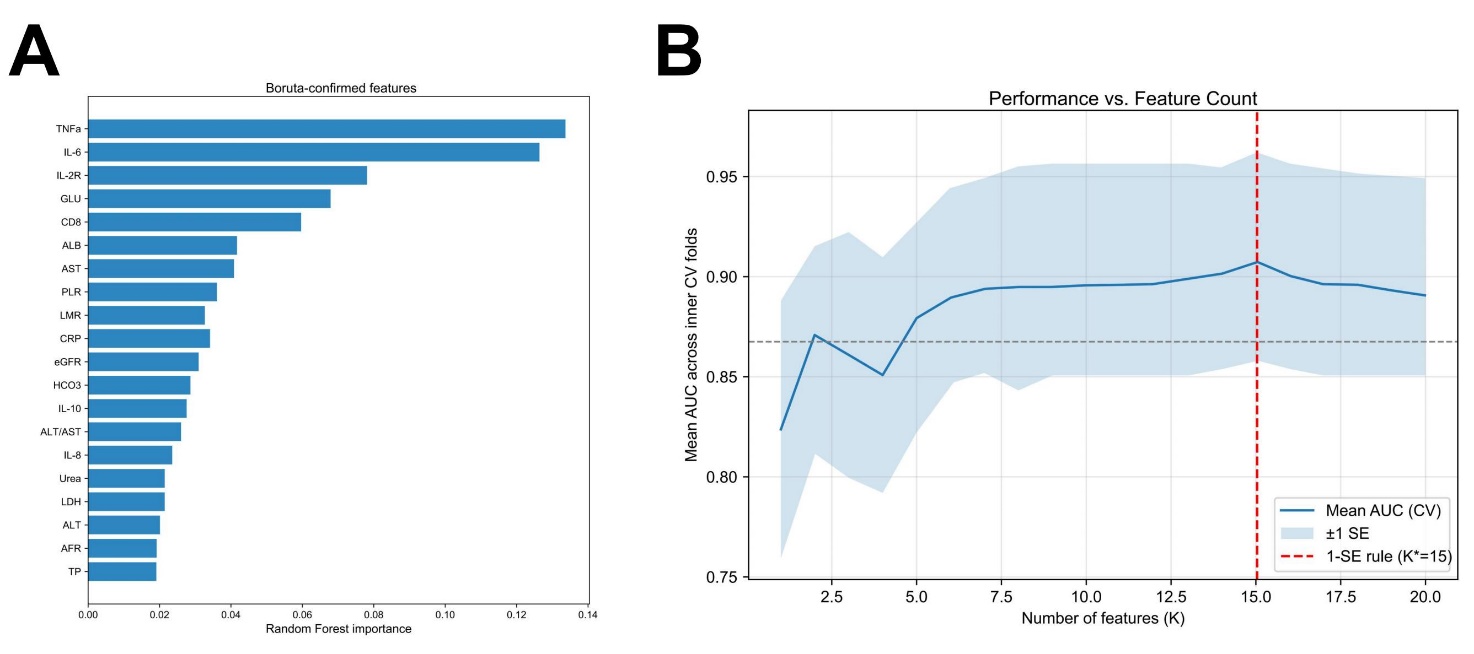


**Figure S2. Boruta feature selection and the relationship between model performance and feature number.** (A) The Boruta algorithm, based on random forest and iterative resampling with shadow feature permutation, was used to identify a robust set of predictive features; (B) Features selected by Boruta were then sequentially added according to their importance, and mean AUC (±1 SE) was calculated through 5×3 repeated cross-validation to generate the performance–feature number relationship curve.


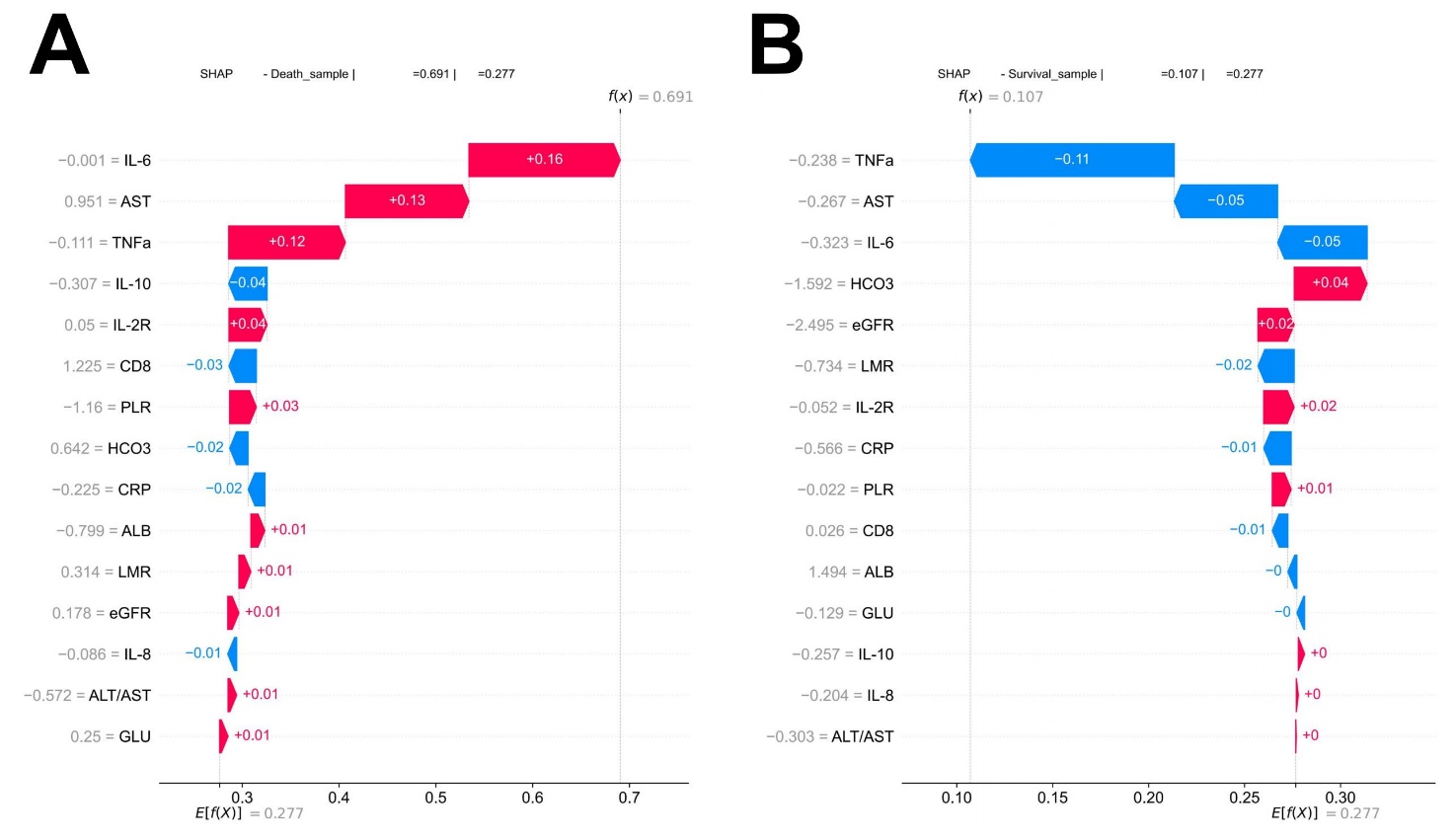


**Figure S3. SHAP-based interpretability analysis of individual-level XGBoost model predictions.** (A) SHAP waterfall plot for a representative high-risk (death) case, illustrating the positive and negative contributions of each feature to the model output; (B) SHAP waterfall plot for a representative low-risk (survival) case, showing the relative influence of each feature on the prediction tendency.


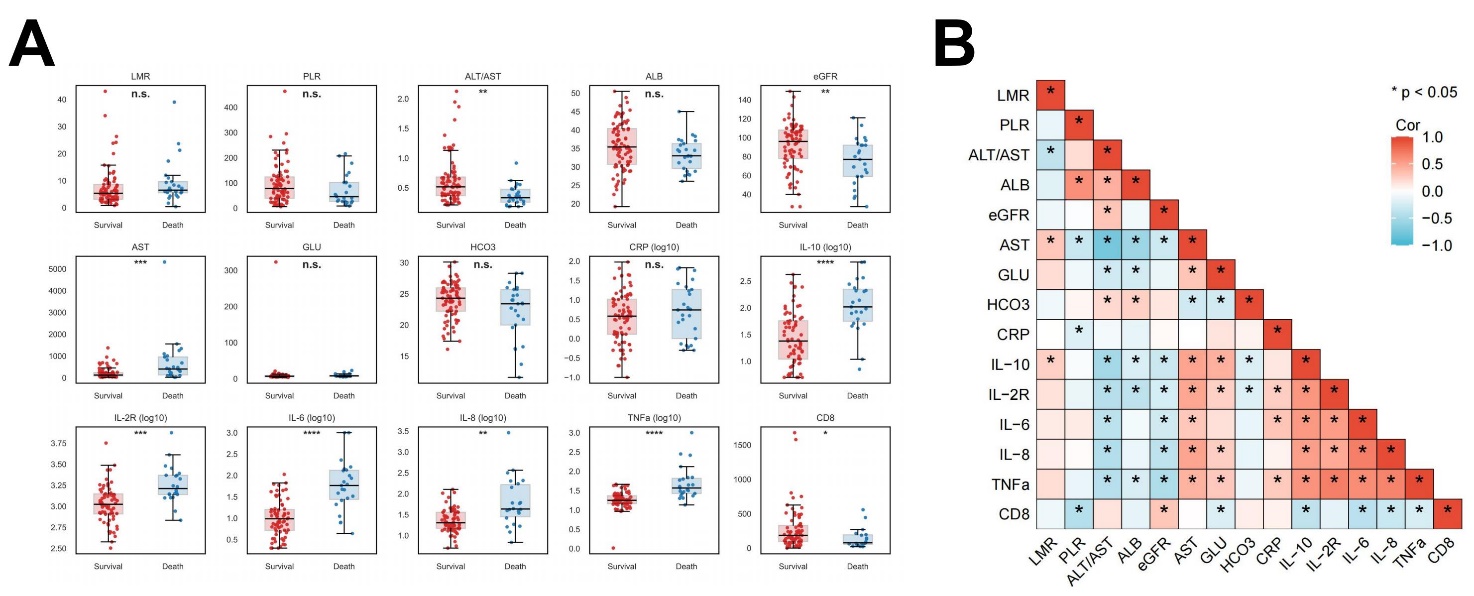


**Figure S4. Group comparison and Spearman correlation analysis of the 15 robust features selected by Boruta.** (A) Group comparisons of the 15 robust features identified and performance-optimized through Boruta selection; (B) Spearman correlation analysis of the 15 features included in the simplified Boruta-XGBoost model.


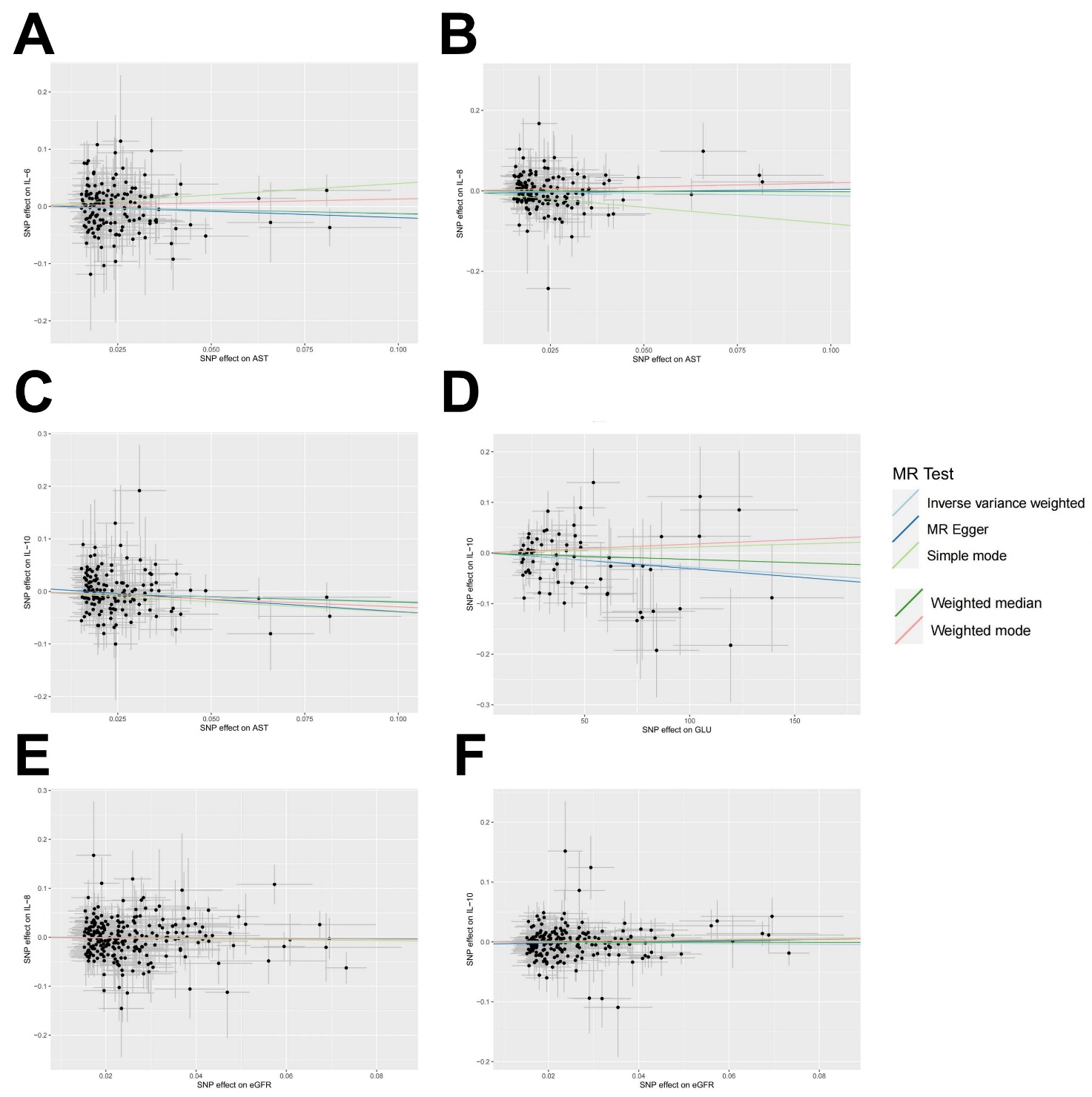


**Figure S5. SNP effect scatter plots for metabolic and inflammatory factor pairs.** (A-F) Scatter plots of SNP effects for AST→IL-6 (A), AST→IL-8 (B), AST→IL-10 (C), GLU→IL-10 (D), eGFR→IL-8 (E), and eGFR→IL-10 (F). Each plot shows SNP effect estimates on exposure (X-axis) and outcome (Y-axis). Five colored lines represent fitted regression lines for five different MR methods.


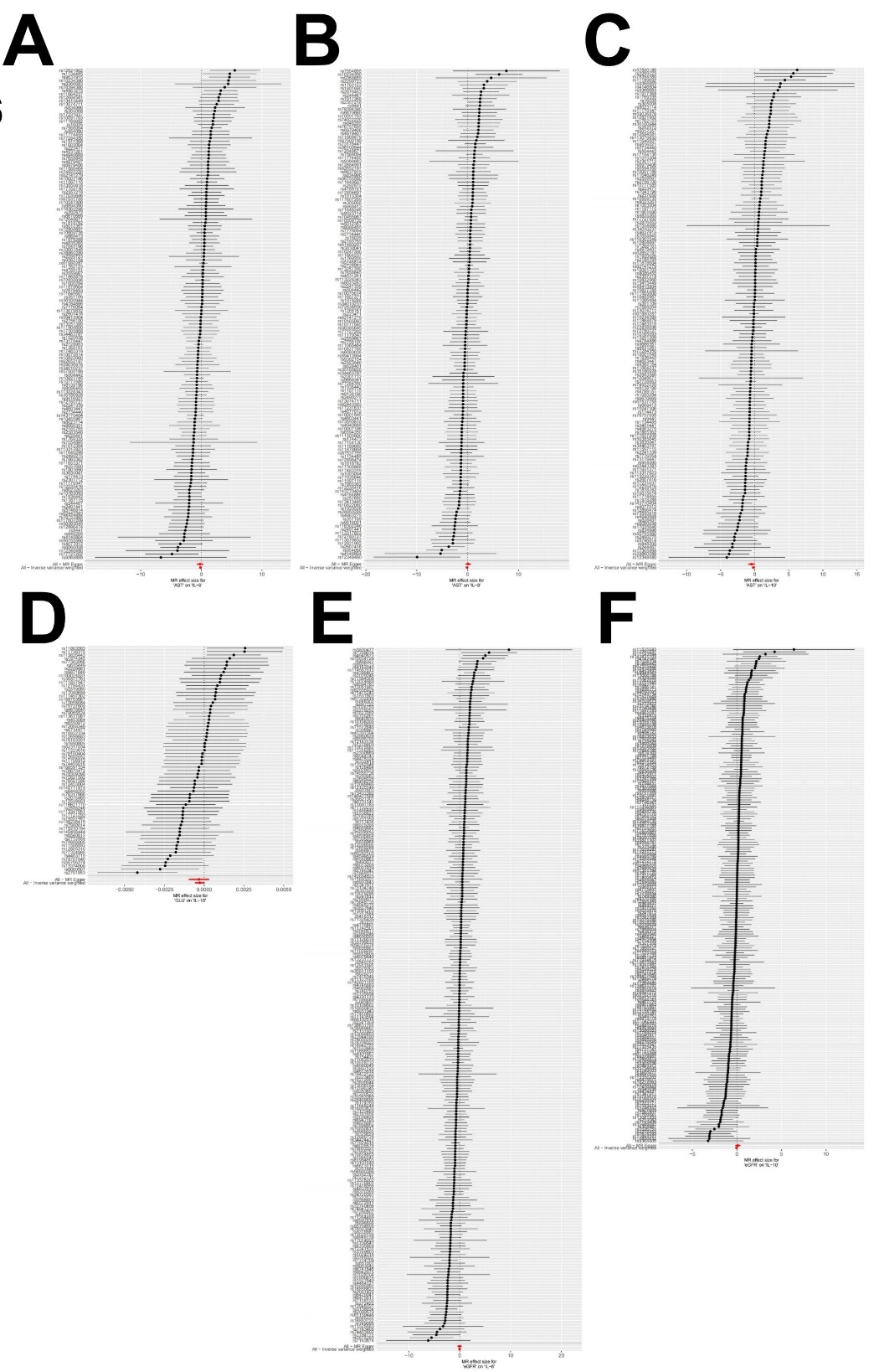


**Figure S6. Forest plots of individual SNP effects.** (A-F) Forest plots of individual SNP effect estimates for AST→IL-6 (A), AST→IL-8 (B), AST→IL-10 (C), GLU→IL-10 (D), eGFR→IL-8 (E), and eGFR→IL-10 (F). Each plot shows the estimated effects and 95% CIs for all SNPs. Combined effect estimates from MR Egger and IVW methods are also shown.


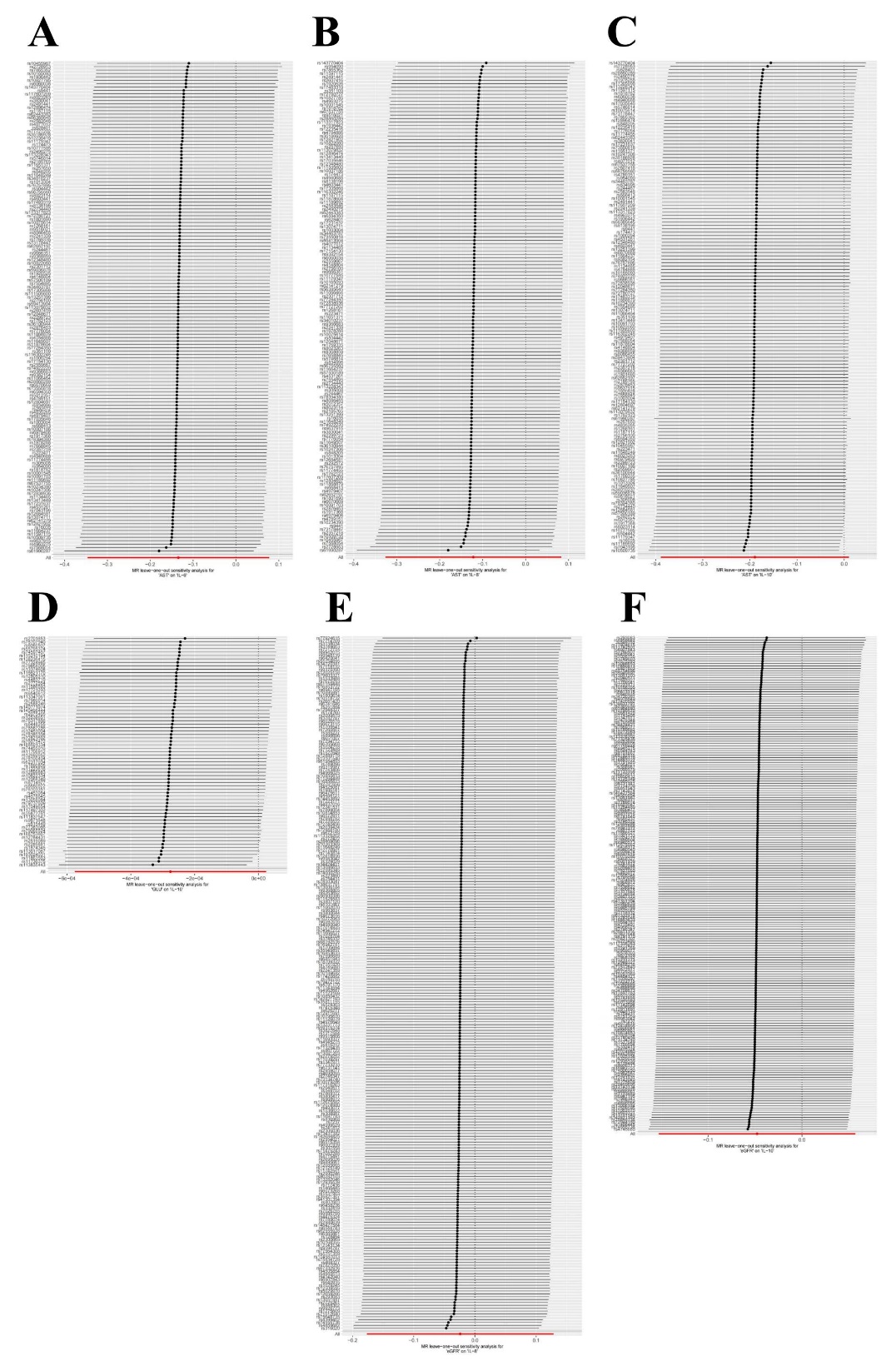


**Figure S7. Leave-one-out sensitivity analysis plots.** (A-F) Leave-one-out sensitivity analysis for AST→IL-6 (A), AST→IL-8 (B), AST→IL-10 (C), GLU→IL-10 (D), eGFR→IL-8 (E), and eGFR→IL-10 (F). The X-axis represents the combined effect estimate after removing one SNP at a time; the Y-axis indicates the excluded SNP.


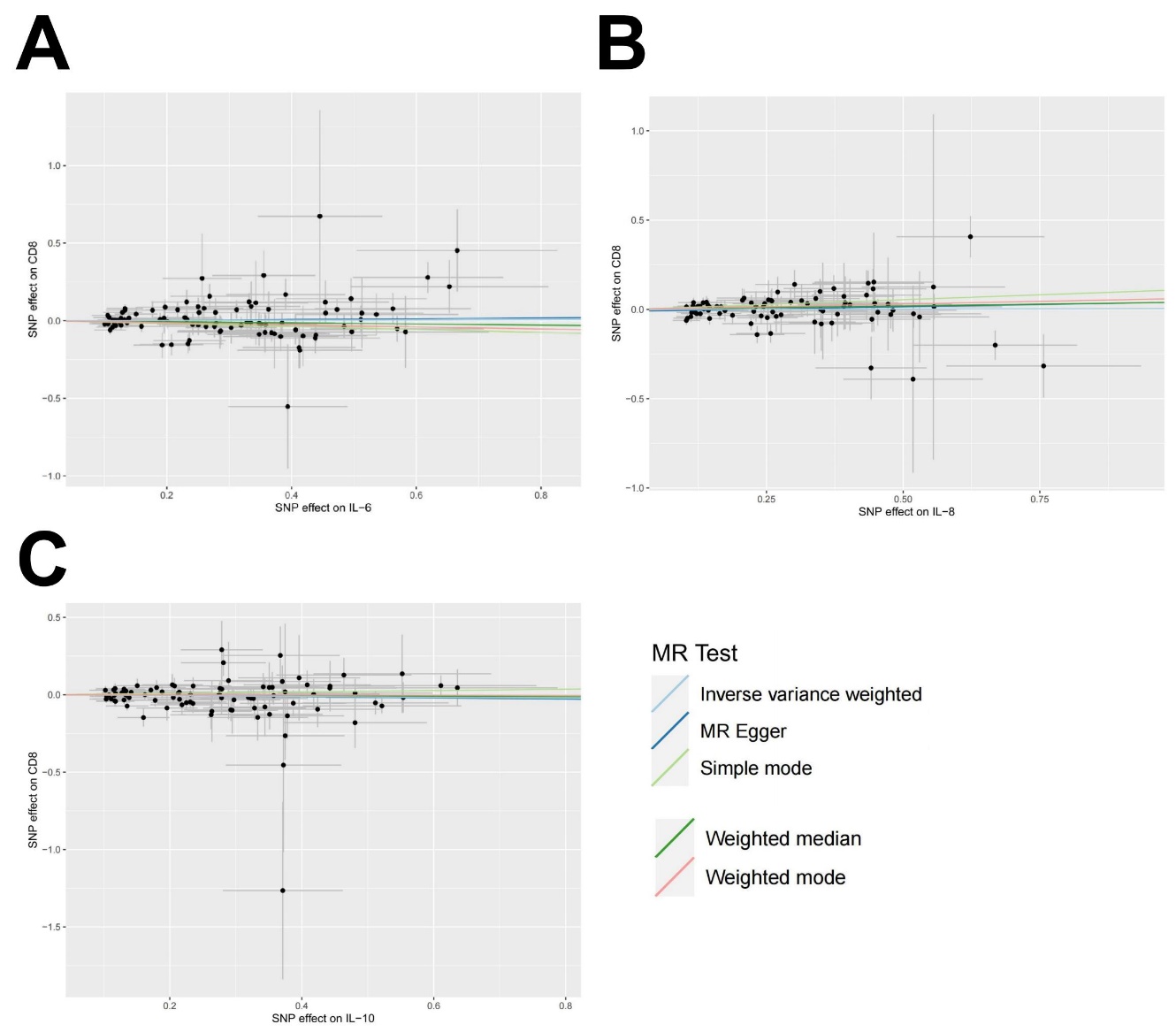


**Figure S8. SNP effect scatter plots for inflammatory and immune factor pairs.** (A-C) Scatter plots of SNP effects for IL-6→CD8 (A), IL-8→CD8 (B), and IL-10→CD8 (C). Each plot shows SNP effect estimates on exposure (X-axis) and outcome (Y-axis). Five colored lines represent fitted regression lines for five MR methods.


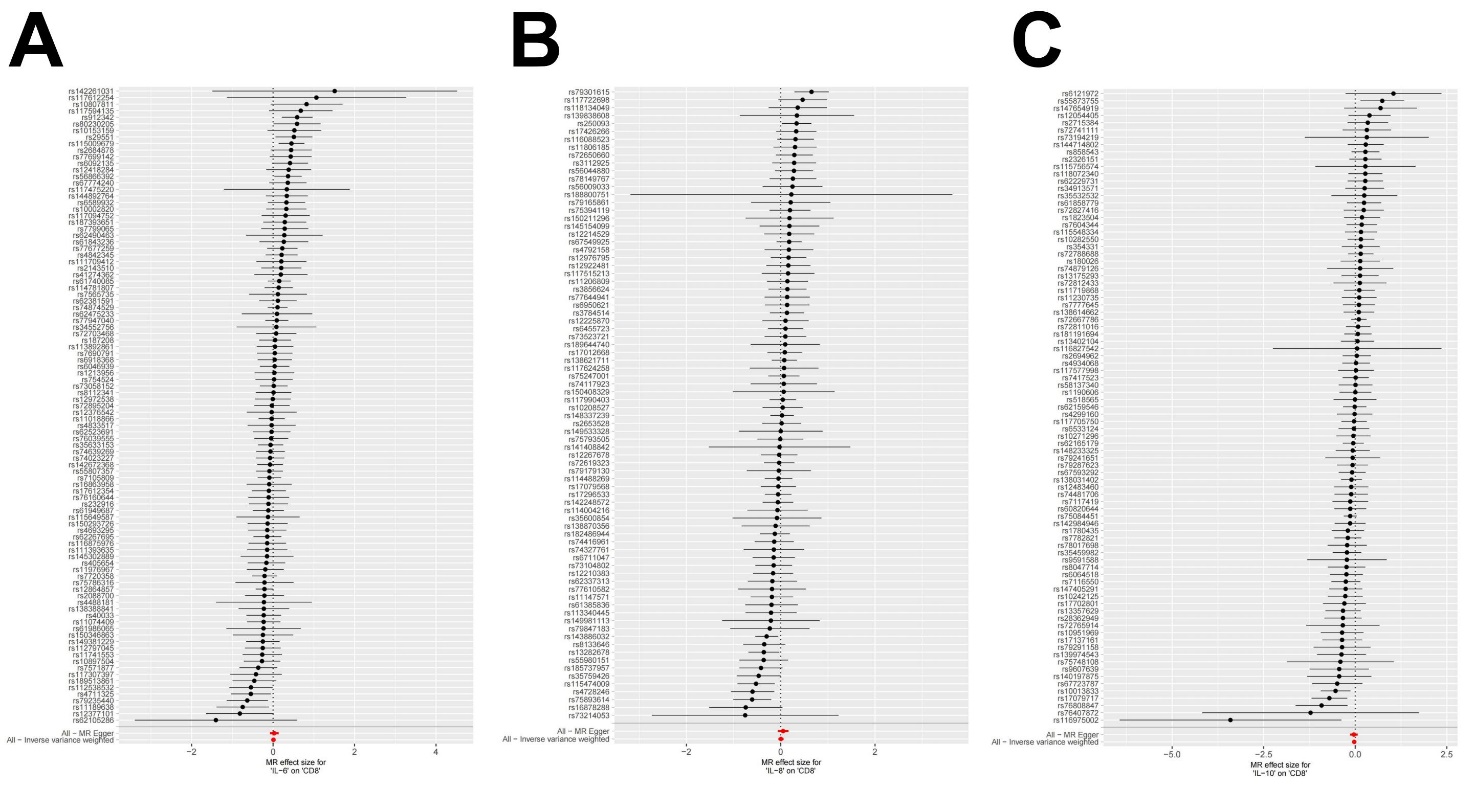


**Figure S9. Forest plots of individual SNP effects.** (A-C) Forest plots of individual SNP effect estimates for IL-6→CD8 (A), IL-8→CD8 (B), and IL-10→CD8 (C). Each plot shows all SNP effect estimates with 95% CIs. Combined effect lines represent the overall estimates from MR Egger and IVW methods.


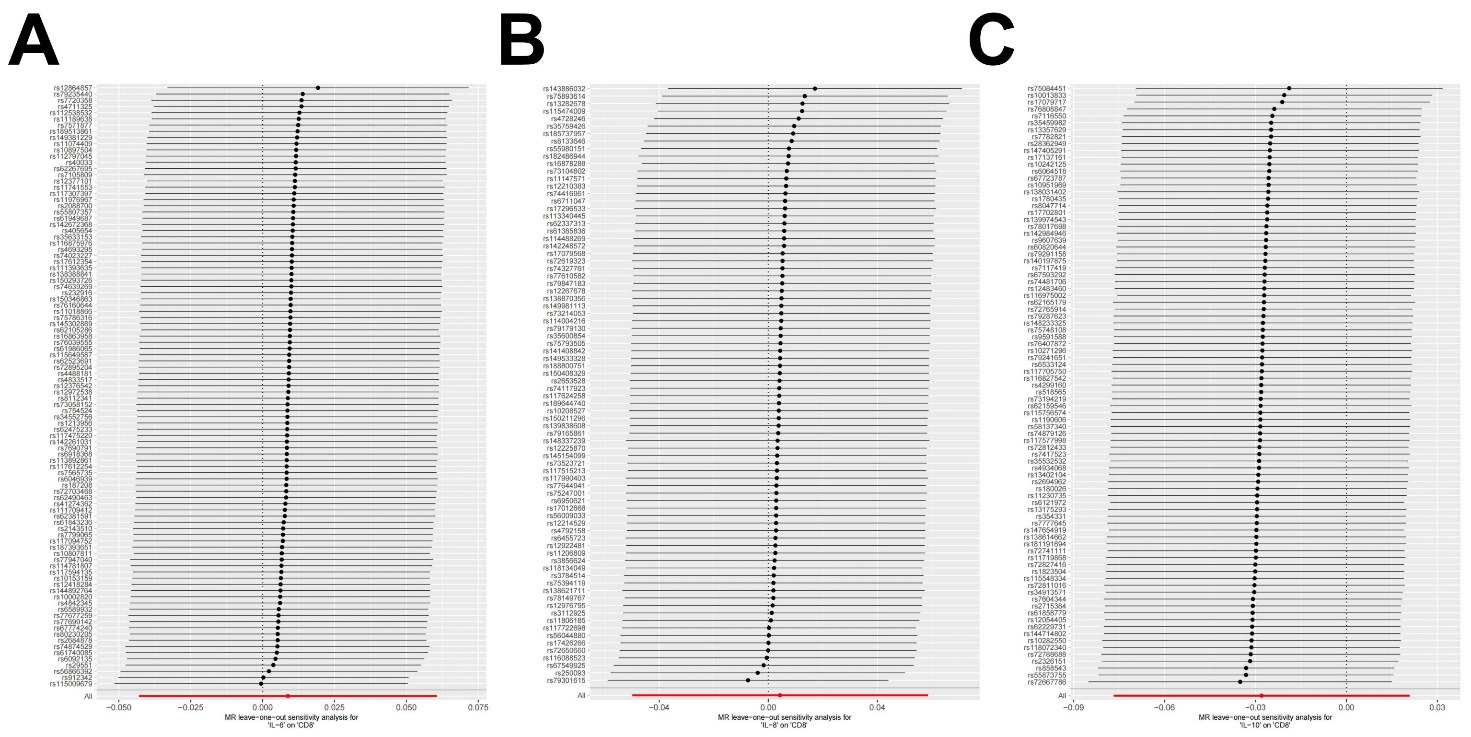


**Figure S10. Leave-one-out sensitivity analysis plots.** (A-C) Leave-one-out sensitivity analysis for IL-6→CD8 (A), IL-8→CD8 (B), and IL-10→CD8 (C). The X-axis shows the combined effect estimate after excluding one SNP at a time; the Y-axis indicates the SNP being excluded.

**Table S1. Heterogeneity analysis results**

| **Outcome** | **Exposure** | **Method** | **Q_pvalue** |
| --- | --- | --- | --- |
| AST | IL-6 | MR Egger | 0.129 |
| AST | IL-6 | Inverse variance weighted | 0.140 |
| AST | IL-8 | MR Egger | 0.427 |
| AST | IL-8 | Inverse variance weighted | 0.430 |
| AST | IL-10 | MR Egger | 0.583 |
| AST | IL-10 | Inverse variance weighted | 0.574 |
| GLU | IL-10 | MR Egger | 0.078 |
| GLU | IL-10 | Inverse variance weighted | 0.091 |
| eGFR | IL-8 | MR Egger | 0.302 |
| eGFR | IL-8 | Inverse variance weighted | 0.318 |
| eGFR | IL-10 | MR Egger | 0.745 |
| eGFR | IL-10 | Inverse variance weighted | 0.5 |
| IL-6 | CD8 | MR Egger | 0.017 |
| IL-6 | CD8 | Inverse variance weighted | 0.019 |
| IL-8 | CD8 | MR Egger | 0.061 |
| IL-8 | CD8 | Inverse variance weighted | 0.061 |
| IL-10 | CD8 | MR Egger | 0.412 |
| IL-10 | CD8 | Inverse variance weighted | 0.440 |

**Table S2. Pleiotropy analysis results**

| **Outcome** | **Exposure** | **Egger_intercept** | **se** | **P value** |
| --- | --- | --- | --- | --- |
| AST | IL-6 | 0.002 | 0.007 | 0.733 |
| AST | IL-8 | -0.006 | 0.007 | 0.357 |
| AST | IL-10 | 0.008 | 0.007 | 0.347 |
| GLU | IL-10 | 0.002 | 0.001 | 0.854 |
| eGFR | IL-8 | 0.0004 | 0.005 | 0.941 |
| eGFR | IL-10 | -0.004 | 0.004 | 0.269 |
| IL-6 | CD8 | -0.004 | 0.010 | 0.663 |
| IL-8 | CD8 | -0.011 | 0.012 | 0.356 |
| IL-10 | CD8 | 0.002 | 0.010 | 0.823 |

Note: se: standard error.

**Table S3. Hyperparameter settings and optimization ranges of machine learning models**

| Model | Parameter | Search Range / Setting | Optimal Value |
| --- | --- | --- | --- |
| Logistic Regression | Solver | lbfgs | lbfgs |
|  | Max Iterations | 1000 | 1000 |
|  | Regularization (C) | [0.01, 0.1, 1, 10, 100] | 1 |
| Random Forest | Number of Estimators | [100, 200, 500, 1000] | 500 |
|  | Max Depth | [3, 5, 7, 9, None] | 7 |
|  | Min Samples Split | [2, 5, 10] | 2 |
|  | Min Samples Leaf | [1, 2, 4] | 1 |
| XGBoost | Learning Rate | [0.01, 0.05, 0.1, 0.2] | 0.05 |
|  | Max Depth | [3, 5, 7, 9] | 5 |
|  | n_estimators | [100, 300, 500] | 300 |
|  | Subsample | [0.6, 0.8, 1.0] | 0.8 |
|  | colsample_bytree | [0.6, 0.8, 1.0] | 0.8 |
|  | scale_pos_weight | [1, 2, 3, 5] | 2 |
| Support Vector Machine (SVM) | Kernel | ['linear', 'rbf'] | rbf |
|  | C | [0.1, 1, 10, 100] | 10 |
|  | Gamma | ['scale', 'auto'] | scale |

Note: Hyperparameter tuning was performed using five-fold, three-repeat stratified cross-validation to maximize the area under the ROC curve (AUC).
